# Effectiveness of relaxation techniques ‘as an active ingredient of psychological interventions’ to reduce distress, anxiety and depression in adolescents: a systematic review and meta-analysis

**DOI:** 10.1186/s13033-022-00541-y

**Published:** 2022-06-28

**Authors:** Syed Usman Hamdani, Zill-e-Huma, Syeda Wajeeha Zafar, Nadia Suleman, Um-ul-Baneen, Ahmed Waqas, Atif Rahman

**Affiliations:** 1 Global Institute of Human Development, Shifa Tameer-e-Millat University, Islamabad, Pakistan; 2 Human Development Research Foundation (HDRF), Rawalpindi, Pakistan; 3 Institute of Population Health, Department of Primary Care and Mental Health, University of Liverpool, Liverpool, UK

**Keywords:** Relaxation techniques, Systematic review, Meta-regression, Anxiety and depression in young people, Active ingredients

## Abstract

**Background:**

Adolescent depression and anxiety are among the leading contributors to health burden worldwide. ‘Relaxation Techniques (RTs)’ are a “set of strategies to improve physiological response to stress” and are frequently cited as an active ingredient of trans-diagnostic, psychosocial interventions for scaling-up care for preventing and treating these conditions in adolescents. However, there is a little evidence on the effectiveness of ‘relaxation techniques’ for this age group.

**Aim:**

As a part of the Wellcome Trust’s Active Ingredients commission, we did a systematic review and meta-analysis to evaluate the effectiveness of RTs to reduce the symptoms of distress, anxiety and depression in young people, aged 14 to 24 years old, globally.

**Methods:**

We searched 10 academic databases to include 65 Randomized Controlled Trials (RCTs) of relaxation-based interventions for young people with the symptoms of anxiety and depression. Primary outcomes were reduction in symptoms of distress, anxiety and/or depression. We employed the Cochrane risk of bias tool and GRADE (Grading of Recommendations, Assessment, Development and Evaluations) guidelines to assess certainty of outcomes pertaining to anxiety, depression and distress. Standardized mean difference was estimated using effect size.

**Results:**

The analysis of 65 RCTs with 8009 young people showed that RTs were highly effective in treating anxiety (pooled effect size of (Standardized Mean Difference-SMD) − 0.54 (95% *CI* − 0.69 to − 0.40); moderately effective in reducing distress (*SMD* = − 0.48, 95% *CI* − 0.71 to − 0.24) and had only a weak effect on improving depression in young people (*SMD* = − 0.28 (95% *CI* − 0.40% to − 0.15). Face-to-face delivered relaxation techniques yielded higher effect size (SMD = − 0.47, 95% CI − 0.64 to − 0.30) compared to online delivery (SMD = − 0.22, 95% CI − 0.48 to 0.04) for anxiety.

**Conclusion:**

Most of the included studies were from High Income Countries (HICs) and had a high risk of bias. Further high-quality studies with low risk of bias, especially from low resource settings are needed to evaluate the evidence for effectiveness of RTs as an active ingredient of psychological interventions to reduce the symptoms of distress, anxiety and depression in young people.

**Supplementary Information:**

The online version contains supplementary material available at 10.1186/s13033-022-00541-y.

## Background

Depression and anxiety disorders contribute significantly to health burden among adolescents worldwide [1, 2]. The published literature reports that most adult mental health problems begin between the ages of 11 and 18 years, with almost half of the mental health problems having started by the age of 14 years [3]. Given the high burden of youth mental health problems [4], evidence-based psychological interventions for prevention and treatment of anxiety and depression in adolescents have been developed [5, 6]; however, it is not fully understood which ‘ingredients’ of these interventions are effective to prevent and treat anxiety or depression in adolescents. Identifying their ‘active ingredients’ can help to understand how these interventions work, specifically which ingredients of these interventions are more effective to reduce or treat anxiety and depression. Unpacking these interventions can contribute to improve the provision of more personalized treatment for child and adolescent mental health problems, globally.

Recently, mental health research initiatives have increasingly focused on developing personalized treatments to improve clinical outcomes by emphasizing the need to understand the mechanisms of mental health interventions and match these interventions strategies to the individual’s needs [7]. There is an urgency and a global push for understanding which ingredients of psychological interventions are more effective and for what mental health problem and developing more "personalized medicine/interventions," particularly among children and adolescents where comparatively little research has been undertaken. To find these next generation of treatment approaches, specifically to transform how mental health problems of adolescents can be understood and addressed, the Welcome Trust, UK launched ‘Active Ingredients’ initiative with the aim to understand the ‘active ingredients (‘building blocks’) of interventions for prevention, treatment, rehabilitation and relapse prevention of adolescent anxiety and depression, globally [8]. By unpacking these interventions, it aims to provide a better understanding of what active ingredients of effective interventions are; how these active ingredients work; through which pathway and for which sub-sample of the participants they might best work [9–11].

### Description of relaxation techniques

Being a low-risk and safe technique, relaxation techniques are frequently cited as an integral component of psychological therapies to manage anxiety and reduce depression [12]. Relaxation techniques are defined as “a set of strategies to improve physiological response to stress” [13]. The underlying treatment goal of all relaxation techniques is to use relaxation to decrease stress or anxiety [12]. There are many types of relaxation techniques including progressive muscle relaxation, relaxation imagery, autogenic training and applied relaxation. These can be administered in many different forms such as a standalone psychological intervention or part of complex therapy in different settings and context and by the very nature of relaxation techniques to improve both physiological and psychological responses to stress, other forms of treatment such as meditation, yoga and tai chi are sometimes classified into the broad category of relaxation techniques [14, 15].

A higher treatment effect of relaxation techniques compared to other psychological interventions including Cognitive Behavioural Therapy (CBT) to improve anxiety and depression [16, 17] have been reported in the literature. Previous meta-analyses show that among all the other types of relaxation therapies, progressive muscle relaxation, relaxation imagery and autogenic training are highly effective in reducing anxiety and depression in adults [12, 18]. In terms of efficacy of relaxation techniques for the specific sub-groups of the sample, findings of meta-analysis conducted by Manzoni et al. [12] showed that in adults there is a consistent and significant effect of relaxation exercises on anxiety. Moreover, these techniques were found to be more effective for young people compared to older adults. Another systematic review by Jorm, Morgan & Hetrick [18] concluded that relaxation exercises were more effective at reducing self-rated depressive symptoms than no or minimal treatment; however, this systematic review also highlighted the need to review the evidence on effectiveness of relaxation techniques to manage anxiety and improve depressive symptoms in adolescents and to include more rigorous research designs such as randomized controlled trials (RCT) in future reviews to ascertain the efficacy of relaxation techniques to improve mental health outcomes.

Although, relaxation techniques have been increasingly included in the mental health intervention packages for scaling-up care for adolescent mental health globally [19–22] and there is evidence for the feasibility and acceptability of relaxation-based interventions with children and adolescents, the relative impact of relaxation based interventions compared with other multiple components of interventions (such as psycho-education, mobilizing social support) to improve mental health outcomes in adolescents still has not been fully explored yet [3]. Moreover, empirical evidence on the efficacy of these interventions to improve child and adolescent mental health outcome is required, particularly in Low and Middle Income Countries (LMICs) [3]. As a part of the Wellcome Trust’s Active Ingredients commission, we studied the role of ‘Relaxation Techniques (RTs)’ as an active ingredient of mental health interventions to reduce distress, anxiety and depression in adolescents with symptoms of depression and anxiety, globally.

### Objectives

The present systematic review and meta-analysis aimed to fill the gap in the literature by evaluating the current evidence on the effectiveness of relaxation techniques, administered individually or in combination with other therapeutic elements, in reducing symptoms of anxiety, distress and/or depression in young people, globally. In keeping with the staging approach to the classification and treatment of mental disorders [23], we included distress as an outcome measure to evaluate the impact of relaxation techniques on distress symptoms in young people. The secondary aim of the current review was to perform moderator analyses to reveal how intervention effect of relaxation techniques may vary across different settings and identify the likely conditions under which relaxation techniques are effective to reduce symptoms of anxiety, distress and/or depression in young people. We studied comparative effectiveness of relaxation techniques across varying delivery models of interventions (i.e., electronic vs face-to-face intervention delivery) to reduce symptoms of anxiety, distress and/or depression in young people.

## Methods

The present review was conducted following the Preferred Reporting Items for Systematic Reviews and Meta-Analyses (PRISMA) guidelines [24]. A protocol for this systematic review was agreed with the Wellcome Trust, UK as the version of record (see Additional file 2: material A).

### Ethics approval

As the current paper is a systematic review of literature and meta-analysis of de-identified published results, no ethical approval to conduct the review was required.

### Study inclusion criteria

The inclusion criteria were formulated in accordance with the PICO criteria. We included all Randomized Controlled Trials (RCTs) in which relaxation technique(s) was used as a standalone intervention or in combination with other elements to prevent and treat symptoms of anxiety and depression among young people aged 14–24.

Our inclusion criteria were;

#### Population

RCTs focusing on young people (aged 14–24 years, as defined by the Wellcome Trust) exhibiting prodromal symptoms of depression, anxiety and distress or near cut-off scores on psychometric scales.

#### Intervention

RCTs where ‘Relaxation Technique(s)’ was used as a standalone intervention or in combination of other elements to reduce the symptoms of distress, anxiety and depression in young people aged 14–24. All modes of intervention delivery were included (e.g., on-site and technology).

#### Control

All types of control arm were eligible to be included.

#### Outcome

RCTs which reported symptoms of distress, anxiety and/or depression as a primary or secondary outcome. Both International Classification of Diseases (ICD)-11 and Diagnostic and Statistical Manual of Mental Disorders, Fifth Edition, Text Revision (DSM-5TR) criteria of diagnoses or symptom severity of distress, anxiety and depression, measured on psychometric scales were considered eligible.

#### Study design

We included individual and cluster randomized controlled trials.

#### Country

Studies from all regions were included.

We excluded studies where;

Intervention was conducted with adolescents who were chronically ill, requiring in-patient care or were with medical comorbidities.

### Search strategy

We searched 10 academic databases including PubMed, Cochrane CENTRAL, PsychInfo, Virtual Health Library, Scopus Open access, Web of Science all databases (Russian database), Psycharticles, Psychextra, Proquest Dissertation and thesis (see Table 1 for search strategy). The search strategy was pilot tested on PubMed. The actual search was conducted between July 7 and July-27, 2020. Four reviewers, working independently from one another, reviewed titles and abstracts, followed by full text screening of eligible studies as per the eligibility criteria. The process of title and abstract screening was aided by the use of Rayyan software [25]. Furthermore, the references of included studies were manually searched and screened to identify and include any relevant trials in the review. Discrepancies in the inclusion process were discussed and resolved in consultation with the senior authors (UH & ZeH).

**Table 1:** Search strategy of peer-reviewed articles

Condition	Search terms
Participants	(teenage” OR “teenagers” OR “teen” OR “teens” OR “youth” OR “young” “youngster” OR” youngsters” OR “young adult” OR “juvenile” OR “adolescent” OR “adolescents” OR “adolescence)
Interventions	"psychological relaxation" OR "mental relaxation" OR "physiological relaxation" OR "therapeutic relaxation" OR "relaxation training" OR "relaxation technique*" OR breathing OR "slow breathing" OR "deep breathing" OR meditation OR "progressive muscle relaxation" OR "imagery" OR "Autogenic training" OR spirituality OR walking OR gardening OR yoga OR "T'ai chi" OR Qigong OR massage OR acupuncture OR "Feldenkrais Method" OR myotherapy OR reflexology OR self-regulation OR autosuggestion OR prayer OR hypnosis OR Pranayama OR biofeedback OR music OR art-therapy OR stress-management OR writing OR exercise OR aromatherapy OR hydrotherapy OR laughing-therapy OR food-therapy OR mindfulness)
Conditions	(“Depression" OR “depressive disorder” OR “depressive symptoms” OR “depressed” OR “anxiety” OR “anxieties” OR “anxiety symptoms” OR “anxiety disorder” OR “anxiety disorders”)
Outcomes	Mental health” OR psychosocial OR “Well-being” OR “self-esteem” OR social OR suicide OR suicidality OR distress OR depress* OR stress OR anxiety OR anxious OR emotional OR internaliz* OR externaliz*
Study design	(“Clinical trial” OR intervention OR trial OR “randomized controlled trial” OR RCT OR “cluster randomized control trial”)
Region	N/A

### Data extraction

Information pertaining to the characteristics of the study sample and interventions was extracted from the included studies. After establishing an adequate inter-rater reliability with senior authors across 20% of the studies, data was extracted by two reviewers for each study. The data from the studies were extracted across three broad matrices: (i) study sample (ii) theoretical underpinnings and components of interventions and (iii) implementation characteristics of interventions. Variables pertaining to the study sample included important characteristics such as age of the study samples, inclusion and exclusion criteria of the studies, and outcome measures. Recognizing the fact that several complex interventions will utilize relaxation techniques as one of many components, we critically reviewed all interventions to deconstruct complex interventions to identify core components of interventions and the terminologies employed to describe them. First, we identified the ‘active ingredients’ of each intervention and type of relaxation techniques used in studies, coded each component and cross-tabulated studies with each component; then we combined these active ingredients with other intervention features and dimensions (e.g., theoretical underpinning, format, dosage and settings). The data on implementation characteristics of the interventions, including measures of intervention fidelity, type of delivery agents and their competency assessment, supervision and training process and resource provision, was also extracted.

### Outcome measures

The primary outcome was reduction in the symptom scores of anxiety or depression or distress, measured at post-intervention, using self-reported, valid and reliable psychometric scales that are scored on a continuum [26]. We included outcome data of scales, reported either as composite scores (total scores) or subscales wise. To estimate a Standardized Mean Difference (SMD), we calculated effect size for each listed primary outcome, using means and standard deviations. In studies, where no data on means and standard deviation was reported, the data on binary outcomes and unadjusted statistical effect sizes was extracted. Using previously-recommended assumptions and formulae [27], these binary outcomes were transformed to *SMD*, based on the assumption that (a) continuous measurements follow a logistic distribution and (b) the variability of the outcomes is the same in both intervention and control groups [28]. For the primary endpoint outcome, we used the end point specified in the studies.

### Data analyses methods

Effect size such as Mean and Standard Deviation (SD) was extracted for continuous outcomes and for binary outcomes, frequency of events and sample size of intervention and control group was extracted. A series of meta-analyses were run, where studies were weighted using random effects model and forest plots were generated exhibiting effect size for each study along with their 95% confidence intervals. Random effects were applied throughout the analyses due to expected clinical, methodological and statistical heterogeneity in the studies. Sensitivity analyses were employed to assess contribution of each study especially outliers to the pooled effect size. Publication bias was assessed using Egger’s regression statistic, where there were more than ten studies. In addition, we also visualized Begg’s funnel plot [29]. If there was a significant publication bias in reporting of an outcome, it was adjusted using the Duval & Tweedie’s trim & fill method [30].

Subgroup analyses were run when specific subgroups (such as theoretical orientation of psychological interventions, type of delivery agents, dosage density of intervention and type of population) were reported in more than 4 studies. We used meta-regression analysis [31] to understand the impact of other therapeutic elements of interventions employed in included studies compared with ‘relaxation techniques’. Meta-regression analysis was run when covariates were reported in more than ten studies [32, 33].

### Risk of bias and quality of evidence

Risk of bias among RCTs was assessed using the Cochrane tool for risk of bias assessments. GRADE (Grading of Recommendations, Assessment, Development and Evaluations) evidence criteria was applied to grade the certainty of evidence for these interventions. Using GRADE profiling method, the strength of evidence for these outcomes was rated from very low to high.

## Results

### Study selection

**Fig. 1:**
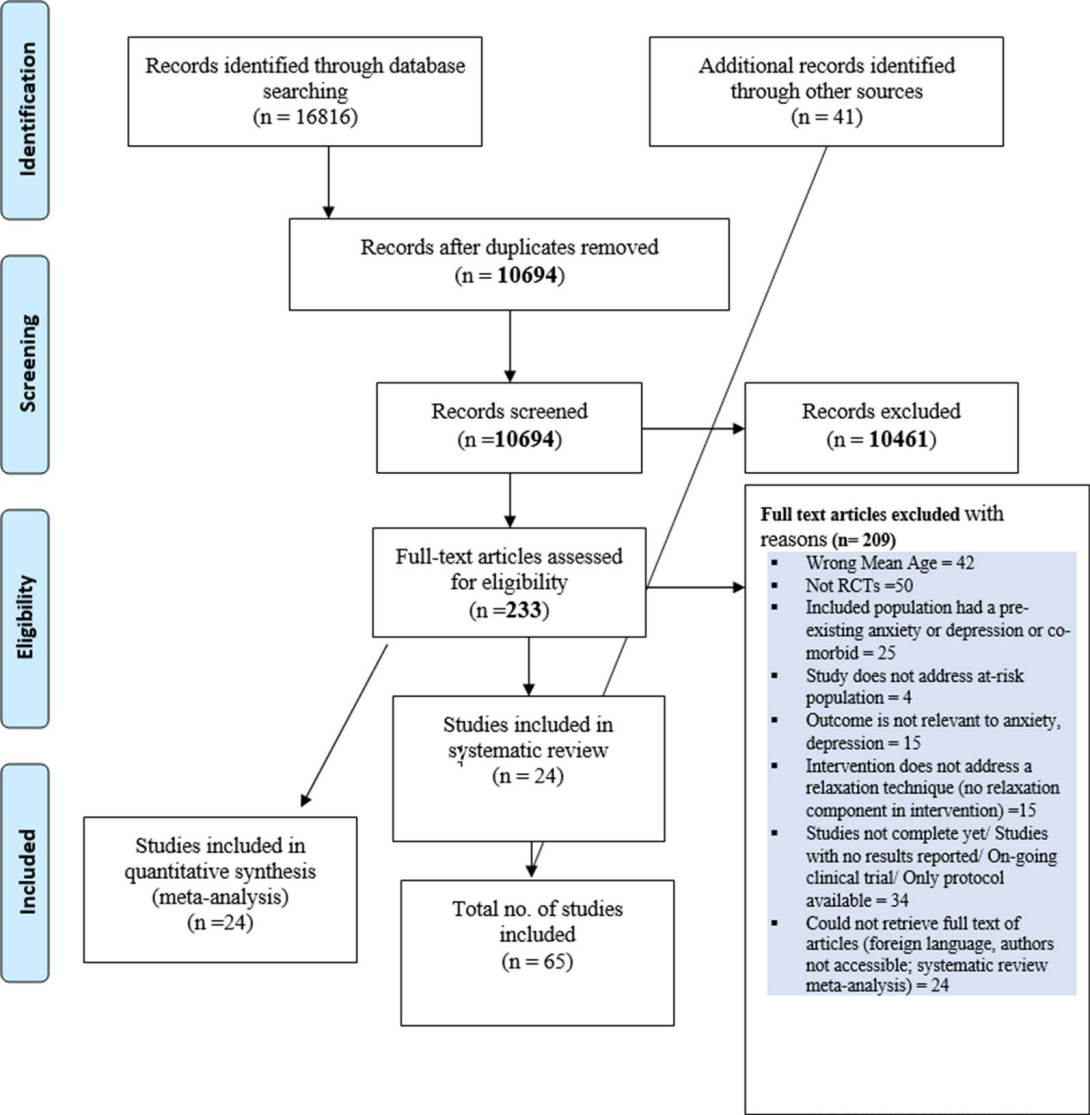
Study selection process

We identified and screened 16,816 records from 10 academic databases. After removing duplicates, 10,694 studies were assessed for title and abstract screening. Out of these, 10,461 studies were excluded as they did not meet the eligibility criteria. Two reviewers independently screened 233 full-text articles and included 24 studies for meta-analysis. We manually searched and screened the references of the primary studies and included 41 more studies for meta-analysis. A total of 65 RCTs with 8009 participants were included in the systematic review and meta-analysis. Figure 1 describes the study selection process.

### Study characteristics

Of the 65 studies, 56 were from High Income Countries, 6 studies were from Upper Middle-Income Countries and only 1 study was from a Lower- and Middle-Income country (i.e., India). In majority of the studies (96%, 62/65), the study participants were students. Mean age of adolescents was 19.09 (± 2.92). In 80% (52/65) of the studies, participants were both male and female adolescents. Studies included adolescents with depression (21%, 14/65), anxiety (9%, 6/65), distress (20%, 13/65), combined symptoms of anxiety, distress and depression (17%, 11/65) and behavioural problems, academic concerns and eating problems (11%, 7/65). In 21% of the studies (14/65), the condition of the study participants was not specified (see Table 2 for the characteristics of the included studies).

**Table 2:** Characteristics of included studies (N = 65)

Sr. No.	Study (Author, year	Country	Sample	Trial Design	Population	Gender/Age, M(SD)	Recruitment setting	Relaxation Type	Clinical outcome
1	(Caldwell et al., 2016)	USA	50	RCT	University students	Both, 18–40 (mean, SD missing)	University	Relaxation	Anxiety
2	(Gold et al., 2017)	Australia	100	cRCT	School students	Both, 13.84 (0.74)	School	Relaxation	Depression
3	(Robledo-Colonia et al., 2012)	Cali, Colombia	74	RCT	Pregnant women	Female, 21 (SD missing)	Prenatal Care Services of three Hospitals	Relaxation	Depression
4	(Harmat et al., 2008)	Hungary	65	RCT	University students	Both, 22.6 (2.83)	University	Relaxation	Depression
5	(Nabkasorn et al., 2006)	Thailand	49	RCT	Nursing students	Female,18.8 (0.7)	Not specified	Relaxation	Depression
6	(Putra et al., 2018)	Indonesia	31	RCT	School students	Female,15–17 (mean, SD missing)	School	Relaxation	Depression
7	(MacMahon & Gross, 1988)	USA	69	RCT	Juvenile	Male, 16.3 (SD missing)	Juvenile detention facilities	Relaxation	Depression
8	(Reynolds & Coats, 1986)	California	21	RCT	School students	Both, 15.65 (SD missing)	School	Relaxation	Depression and anxiety
9	(Roth, 1989)	USA	80	RCT	College students	Both, 20.8 (SD missing)	College	Relaxation	Depression and anxiety
10	(Roth & Holmes, 1987)	UK	57	RCT	College students	Both, 18.9 (1.3)	College	Relaxation	Depression and anxiety
11	(Velasquez et al., 2015)	Columbia	125	RCT	School age children	Not specified	Not specified	Relaxation	Depression and anxiety
12	(Norris et al., 1992)	UK	91	RCT	School students	Both, 16 (SD missing)	School	Relaxation	Depression anxiety and stress
13	(Walsh et al., 2016)	USA	64	RCT	Students	Female, 19.15 (SD missing)	Not specified	Multicomponent	Depression
14	(Raes et al., 2014)	Belgium	335	cRCT	School students	Both, 13- 20 (mean, SD missing)	School	Multicomponent	Depression
15	(Sagon et al., 2018)	USA	103	RCT	Freshmen university students	Both, 18.15 (.46)	University	Multicomponent	Depression
16	(Stasiak et al., 2014)	New Zealand	34	Pilot RCT	School students	Both, 13–18 (mean, SD missing)	School	Multicomponent	Depression
17	(Cui et al., 2016)	China	120	RCT	College students	Both, 19.42 (1.66)	College	Multicomponent	Depression
18	(Felver et al., 2015)	USA	47	RCT	School students	Both, 15 (SD missing)	School	Multicomponent	Depression
19	(Khalsa et al., 2012)	USA	100	RCT	School Students	Both, 16.8 (0.6)	School	Multicomponent	Stress
20	(De Vibe et al., 2013)	Norway	288	RCT	University students	Both, 23 (SD missing)	University	Multicomponent	Stress
21	(De Vibe et al., 2013; Erogul et al., 2014)	New York, USA	57	RCT	Medical students	Both, 23.5 (1.7)	College	Multicomponent	Stress
22	(Phang et al., 2015)	Malaysia	75	RCT	Medical students	Both, 21.14 (1.10)	College	Multicomponent	Stress
23	(Scholten et al., 2016)	Netherlands	138	RCT	School students	Both, 13.90 (.91)	School	Multicomponent	Anxiety
24	(Grassi et al., 2011)	Not specified	75	RCT	University students	Female, 20.86 (1.27)	University	Multicomponent	Anxiety
25	(Dvořáková et al., 2017)	USA	107	Pilot RCT	College students	Both, 18.2 (0.4)	College	Multicomponent	Depression and anxiety
26	(Vázquez et al., 2012)	Spain	133	RCT	University students	Both, 23.3 (SD missing)	University	Multicomponent	Depression and anxiety
27	(McGrady et al., 2012)	USA	105	RCT	First year medical students	Both, age not specified	Not specified	Multicomponent	Depression and anxiety
28	(Levin et al., 2017)	USA	79	RCT	College students	Both, 21.61(5.48)	College	Multicomponent	Depression and anxiety
29	(Blake et al., 2018)	Australia	123	RCT	School students	Both, 14.48 (SD missing)	School	Multicomponent	Depression and anxiety
30	(Merry et al., 2012)	New Zealand	187	RCT	School student	Both, 15.6 (SD missing)	School	Multicomponent	Depression and anxiety
31	(Seligman et al., 2007)	USA	212	RCT	First year undergraduate students	Both, age not specified	College	Multicomponent	Depression and anxiety
32	(Kenardy et al., 2003)	Australia	74	RCT	University students	Both, 19.92 (4.78)	University	Multicomponent	Depression and anxiety
33	(Seligman et al., 2000)	USA	225	RCT	University students (1st year under-graduates)	Both, age not specified	University	Multicomponent	Depression and anxiety
34	(Calear et al., 2009)	Australia	1477	cRCT	School students	Both, 14.34 (0.75)	School	Multicomponent	Depression and anxiety
35	(Chen et al., 2013)	China	60	RCT	Nursing students	Both, 19.5(0.87)	University	Multicomponent	Depression and anxiety
36	(Delgado et al., 2010)	Not specified	32	RCT	University students	Female, 18–24 (mean, SD missing)	University	Multicomponent	Depression and anxiety
37	(Shapiro et al., 1998)	USA	78	RCT	Medical students	Both, age not specified	University	Multicomponent	Depression and anxiety
38	(Astin, 1997)	USA	28	RCT	University students	Both, age not specified	University	Multicomponent	Depression and anxiety
39	(Shearer et al., 2015)	USA	46	RCT	University students	Both, age not specified	University	Multicomponent	Depression and anxiety
40	(Hilyer et al., 1982)	USA	43	RCT	School students	Male, 15–18 (mean, SD missing)	School	Multicomponent	Depression and anxiety
41	(Melnyk et al., 2009)	USA	47	RCT	Adolescents	Both, 14–16 (mean, SD missing)	Not specified	Multicomponent	Depression and anxiety
42	(Ștefan et al., 2018)	Romania	46	RCT	College students	Female, 18.92 (SD missing)	College	Multicomponent	Anxiety and stress
43	(Chiauzzi et al., 2008)	USA	157	RCT	College students	Both, 18–24 (SD missing)	College	Multicomponent	Anxiety and stress
44	(Saravanan & Kingston, 2014)	Malaysia	66	RCT	Medical students	Both, 19 (1.04)	University	Multicomponent	Anxiety and stress
45	(Fleming et al., 2012)	New Zealand	30	RCT	School students	Male, 14.9 (.79)	School	Multicomponent	Anxiety and stress
46	(Deckro et al., 2002)	USA	90	RCT	College students	Both, 24 (SD missing)	College	Multicomponent	Anxiety and stress
47	(Nguyen-Feng et al., 2017)	USA	243	RCT	College students	Both, 18–21 (mean, SD missing)	College	Multicomponent	Depression, Anxiety and stress
48	(Zhang et al., 2018)	China	62	RCT	University students	Both, 18.41(2.01)	University	Multicomponent	Depression and stress
49	(Bluth et al., 2016)	North Carolina ( USA)	23	RCT	School students	Both, 16.8 (1.3)	School	Multicomponent	Depression, anxiety and stress
50	(Flett et al., 2019)	New Zealand	208	RCT	University students	Not specified, 20.08(2.8)	University	Multicomponent	Depression, anxiety, and stress
51	(Song & Lindquist, 2015)	South Korea	44	RCT	Undergraduate nursing students	Both, 19.6 (1.7)	Not specified	Multicomponent	Depression, anxiety, and stress
52	(Rentala et al., 2019)	India	209	RCT	College students	Female, 16–19 (SD missing)	College	Multicomponent	Depression, anxiety and stress
53	(Hall et al., 2018)	China	54	RCT	University students	Both, 22.30 (2.63)	University	Multicomponent	Depression, anxiety, and stress
54	(Hindman et al., 2014)	USA	34	RCT	University students	Both, 22.35 (SD missing)	University	Multicomponent	Depression, anxiety, and stress
55	(Levin et al., 2019)	USA	39	Pilot trial RCT	University students	Both, 20.51 (2.73)	College	Multicomponent	Depression, anxiety, and stress
56	(Ellis et al., 2011)	Australia	26	RCT	University students	Both, 19.67 (1.66)	University	Multicomponent	Depression, anxiety, and stress
57	(Gallego et al., 2015)	Spain	53	RCT	University students	Both, 20.07 (SD missing)	University	Multicomponent	Depression, anxiety and stress
58	(Berger et al., 1988)	USA	232	RCT	College students	Both, 20 (SD missing)	College	Multicomponent	Depression anxiety and stress
59	(Van Aubel et al., 2020)	Neatherland	55	RCT	General population	Both, 21.36 (2.39)	Community	Multicomponent	Depression and Anxiety
60	(Nguyen-Feng et al., 2016)	USA	314	RCT	College students	Both, 18–21 (mean, SD missing)	College	Multicomponent	Depression, anxiety and stress
61	(Warnecke et al., 2011)	Australia	84	RCT	University students	Both, 23.92 (3.2)	University	Multicomponent	Depression and Anxiety
62	(Moir et al., 2016)	New Zealand	402	RCT	University students	Both, 21 (SD missing)	University	Multicomponent	Depression and Anxiety
63	(Nguyen-Feng et al., 2019)	USA	382	RCT	College students	Both, 21.3 (SD missing)	College	Multicomponent	Depression and Anxiety
64	(Levin et al., 2016)	USA	234	RCT	University students	Both, 20.51(2.73)	University	Multicomponent	Depression and Anxiety
65	(Hazlett-Stevens & Oren, 2017)	USA	92	RCT	College students	Both, 22.1 (4.7)	College	Multicomponent	Depression and Anxiety

_RCT: Randomized Controlled Trial (RCT); cRCT: Cluster Randomized Controlled Trial_

### Interventions’ characteristics

Of the 65 studies, 12 (18%) studies reported ‘relaxation technique/s’ as a standalone intervention to reduce symptoms of anxiety, distress and depression in adolescents. The most commonly reported ‘relaxation techniques’ in the literature were Progressive Muscle Relaxation Techniques-PMR, breathing, exercise, walking meditation, stretching, relaxation imagery and meditation. In 82% (53/65) of the studies relaxation techniques were implemented as an integral component of other psychotherapies, multicomponent interventions such as Cognitive Behavioural Therapy (CBT), Mindfulness and Acceptance and Commitment Therapy (ACT). Other components of multicomponent interventions included identifying affect, psychoeducation and mindfulness exercises. A list of ‘relaxation techniques’ reported in the studies are mention in Table 3. The detail on characteristics of included interventions is mentioned in Additional file 2: Table S6).

**Table 3:** List of relaxation techniques used in included studies (N = 65)

Category of relaxation technique
Progressive Muscle Relaxation (PMR)
Breathing
Exercise
Walk
Stretches
Relaxation (music, art)
Autogenic training
Meditation (sitting, eating, walking)

Interventions in 34/65 (52%) studies were delivered in an educational setting (school/colleges/universities). Relaxation techniques (either as a standalone or in combination with other elements) were delivered in group format in 57% (37/58) of the studies. Among the studies reporting relaxation techniques as a standalone intervention, the mean number of sessions was 22.27 (± 12.48), over 9.55 (± 2.92) weeks. Mean session duration was 66.5 (± 32.32) minutes. Out of 12 studies, two studies did not report program duration; one study was of 2 h and one study did not report session duration. The average program duration for multicomponent interventions was 7.39 (± 6.35) weeks; average number of sessions were 9.04 (± 8.40) and average session duration was 72.14 min (± 37.61). Among studies using multicomponent interventions, nine studies did not report program duration; one study was of 2 h, three studies did not report session duration and 11 studies did not report session duration. Booster sessions were delivered in 3 trials. In 37/65 (57%) studies, relaxation interventions (either as a standalone or in combination with other elements) were delivered by the specialists. Relaxation based interventions were self-administered in 18/65 (28%) studies and the intervention was delivered online in 4/65 (6%) studies. In 62/65 studies, mental health was reported as a primary outcome.

### Outcomes

Different outcome measures were reported to measure anxiety, distress and depression in the included studies. In majority of the studies (n = 12) Depression, Anxiety and Stress Scale (DASS) was used to measure all three outcomes (distress, anxiety and depression). The outcome measures used for anxiety were Spence Children’s Anxiety Scale (SCAS) (n = 3), The Spielberger State-Trait Anxiety Inventory (STAI) (n = 3), State Trait anxiety (STAI) (n = 3), Anxiety Sensitivity Index (n = 3) and Beck Anxiety Inventory (BAI) (n = 2). Many studies used several other tools for anxiety (n = 20). The outcome measures used for depression were Beck Depression Inventory (BDI) (n = 9), Centre for Epidemiologic Studies Depression Scale (CES-D) (n = 7), Reynolds’ Adolescent Depression Scale (RADS) (n = 4), Patient Health Questionnaire (PHQ-9) (n = 3), Child Depression Rating Scale Revised (CDACR) (n = 3). Moreover, various studies used different types of tools to measure depression (n = 12). Outcome measures used to measure distress were Perceived Stress Scale (n = 13), Penn State Worry Questionnaire (PSWQ) (n = 3), Kessler Perceived Distress Scale (n = 2), and General Health Questionnaire (GHQ) (n = 2). Other studies used different tools to measure distress (n = 3) (list of outcome measures used in studies is given in Additional file 2: Table S8).

### Control groups

In the included studies, relaxation techniques were compared with control groups, including wait-list controls (n = 16), treatment-as-usual (n = 7), no active intervention (n = 23), placebo control (n = 1) or active controls (n = 18).

### Effect of relaxation-based interventions to reduce symptoms of anxiety

**Fig. 2:**
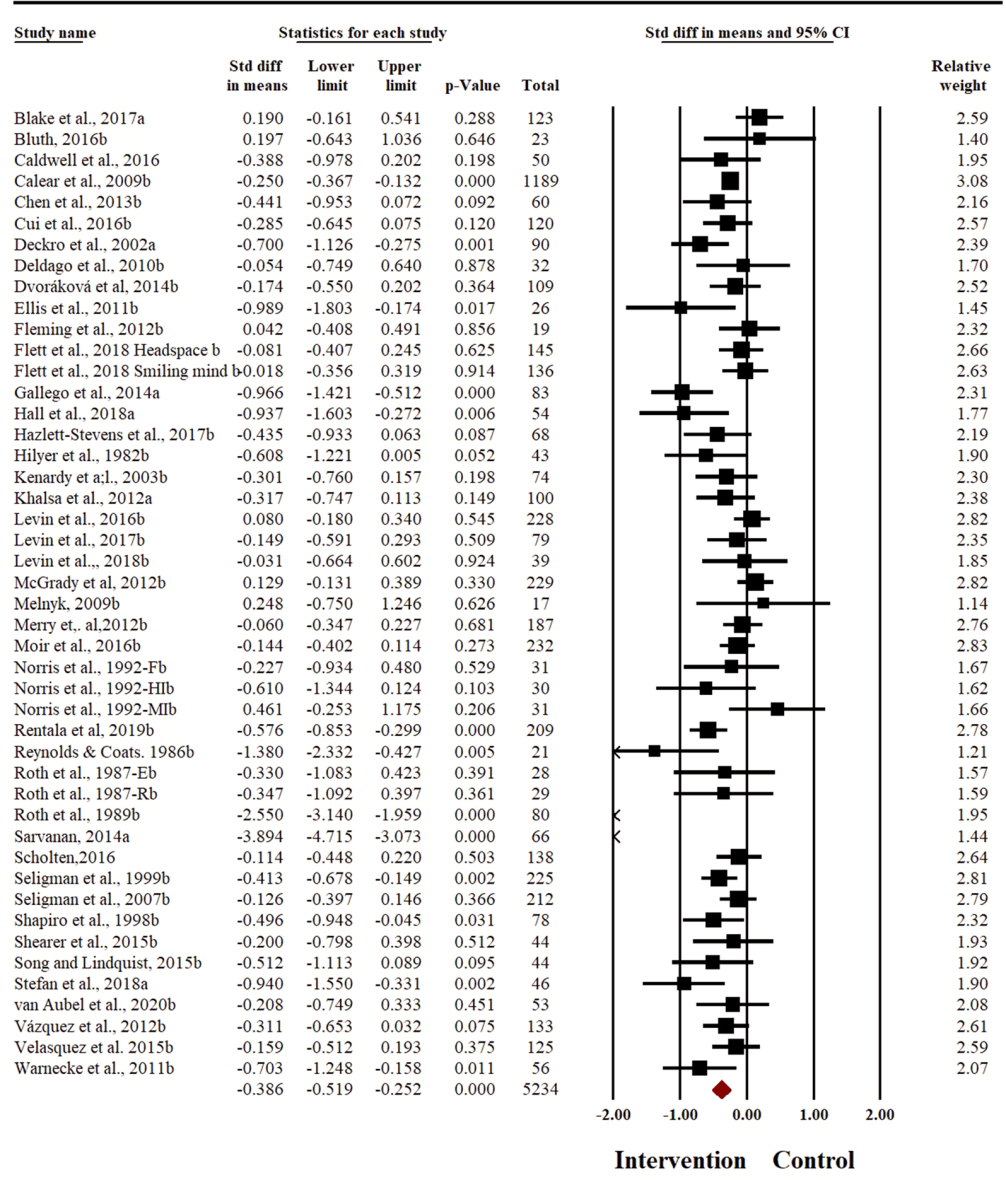
Forest plot for anxiety outcome

Meta-analysis was conducted with 8009 participants. Effectiveness of relaxation-based interventions in the treatment of anxiety was explored in 46 studies, with a cumulative sample size of 5234 participants (2486 in intervention and 2748 in control arm). Meta-analysis with random effects model showed that relaxation techniques were effective to reduce the symptoms of anxiety in adolescents at post-intervention (*SMD* − 0.386, 95% *CI* − 0.52 to − 0.25) (Fig. 2). There was evidence for substantial heterogeneity across the studies (*I*^*2*^ = 79.16%, *t*^*2*^ = 0.15). No significant changes in pooled effect size were observed on sensitivity analysis. Egger’s regression statistic was significant (*t* = 2.32, *p* = 0.02, see Additional file 2: Fig. S5), demonstrating significant publication bias in reporting of anxiety outcome. The pooled effect size increased after adjusting for the publication bias (*SMD*− 0.54, 95% *CI* − 0.69 to − 0.40).

### Effect of relaxation-based interventions to reduce symptoms of distress

**Fig. 3:**
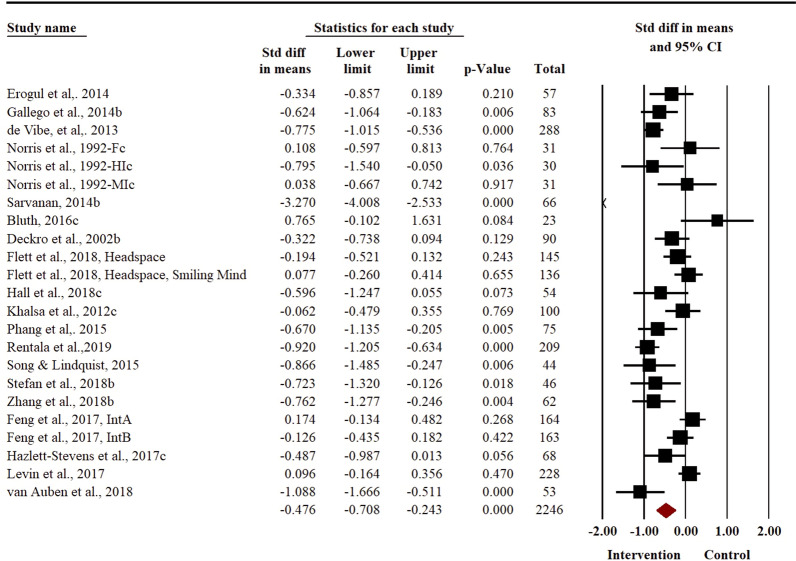
Forest plot for distress outcome

Distress was reported as an outcome in a total of 23 studies, with a cumulative sample size of 2246 (1122 participants in intervention arm and 1124 participants in control arm). There was a substantial heterogeneity in reporting of this outcome (*I*^*2*^ = 85.08%, *t*^*2*^ = 0.26). Meta-analysis revealed a moderate effect size in favour of the intervention group (*SMD* = − 0.48, 95% *CI* − 0.71 to − 0.24) (Fig. 3) to reduce the symptoms of distress in adolescents. Removal of outlier studies in sensitivity analyses did not reveal any significant changes in the pooled effect size for distress outcome. Egger’s regression statistic revealed a non-significant publication bias in reporting of distress outcome (*P* = 0.30, see Additional file 2: Fig. S6).

### Effect of relaxation-based interventions to reduce symptoms of depression

**Fig. 4:**
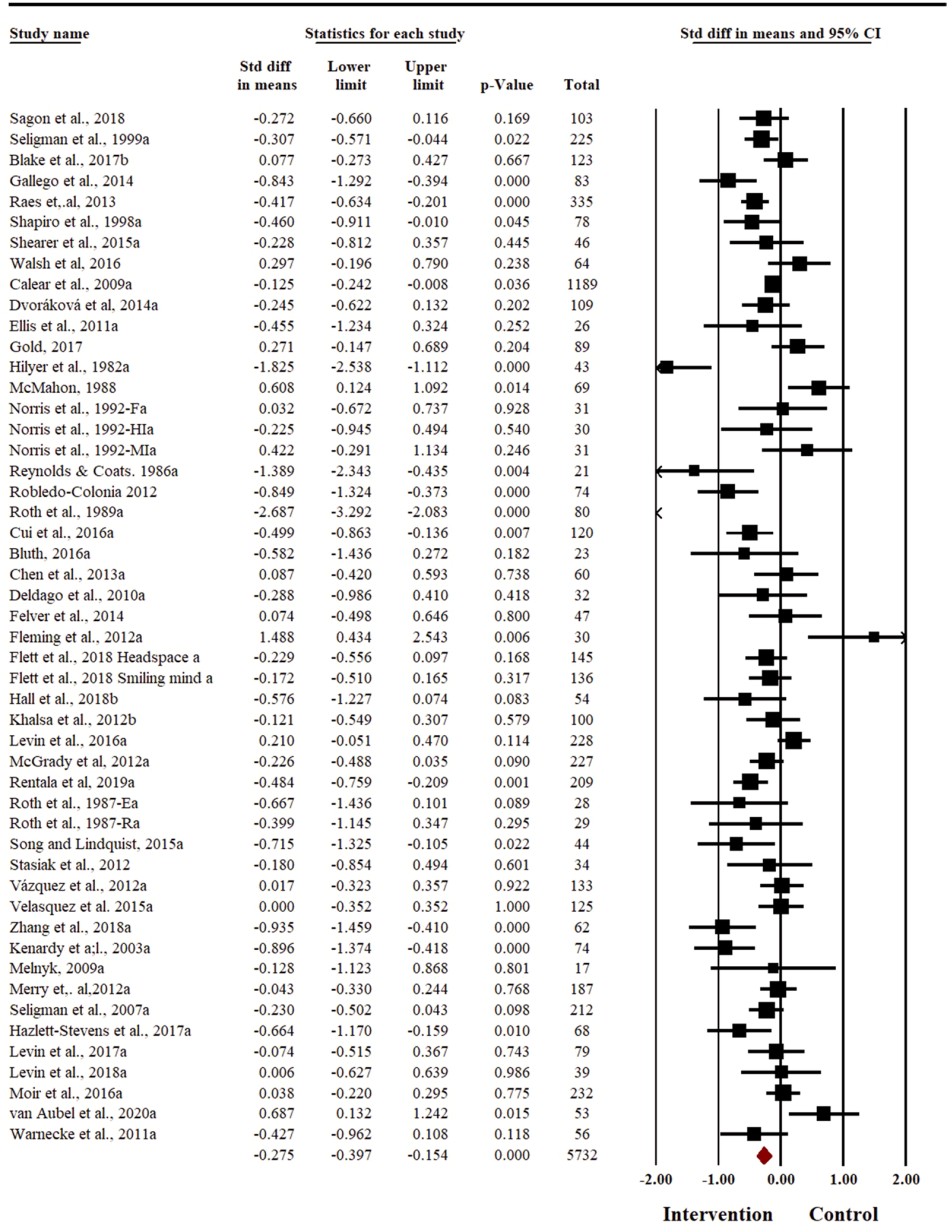
Forest plot for depression outcome

Depression was reported as an outcome in a total of 50 studies, with a cumulative sample size of 5732 participants (2719 in intervention arm and 3013 in control arm). There was small evidence to support relaxation techniques being effective to reduce the symptoms of depression in adolescents (*SMD* = − 0.28 (95% *CI* − 0.40% to − 0.15) (Fig. 4). There was a substantial heterogeneity in reporting of depression outcome (*I*^*2*^ = 76.82%, *t*^*2*^ = 0.13). The sensitivity analysis did not result in any change in the pooled effect size for depression outcome. There was no evidence of publication bias (Egger’s regression *p* = 0.36, see Additional file 2: Fig. S7).

### Sub-group and moderator analyses

We did a sub-group and meta-regression analyses to evaluate in which settings relaxation techniques work to reduce the symptoms of anxiety, distress and/or depression in adolescents. *Anxiety: *Subgroup analyses revealed that face-to-face, individually delivered, multicomponent interventions with a booster session yielded a higher effect size to reduce the symptoms of anxiety in adolescents. However, these results were statistically non-significant (Table 4). Data regarding intervention dosage (including number of sessions; duration of sessions and overall programme) was provided in 30 studies reporting anxiety outcome. To analyse association of dosage of intervention with improvement in the symptoms of anxiety (effect size) meta-regression analyses were run, after removing three of the outlier studies [34–36]. The density of intervention dosage explained 44% of the variation in overall effect size (Table 5) (Additional file 2: Figs. S8–S10). Age of participants explained 13% of variance in anxiety outcome, where age associated inversely with effect size (*B* =  −  0.07, *SE* = 0.03, *p* = 0.04). *Depression:* Subgroup analyses did not reveal any significant subgroup differences based on the mode and format of intervention delivery, types of intervention and booster sessions for depression outcome (Table 4). A total of 33 studies reported statistics pertaining to density of dosage of interventions. The moderator analysis of the data revealed that characteristics of ‘relaxation techniques’ (format, dosage, components) did not have any effect to reduce the symptoms of depression in adolescents. Multivariate meta-regression analyses revealed that 5% of the variation in effect size was explained by density of dosage of interventions. None of the indicators of the dose of interventions reached statistical significance (Table 5, Additional file 2: Figs. S11–S13). Age did not yield any significant association with effect size, explained only 4% of variance in the depression outcome (*B* = -0.04, *SE* = 0.04, *p* = 0.33). *Distress:* Subgroup analyses revealed that studies with low risk of bias, multicomponent, individually delivered face-to-face interventions, with a booster dose were more effective in reducing the symptoms of distress. Only mode of delivery and booster dose were found to be statistically significant (Table 4). Multivariate regression model pertaining to density of intervention dosage of explained 47% of variation in effect size for distress outcome. None of the indicators of dose of interventions emerged as a significant predictor in this model (Table 5, Additional file 2: Figs. S14–S16). The results remained consistent even after removal of the outlier study [36]. Age did not yield any significant association with effect size, explained only 1% of variance to reduce the symptoms of distress (*B* = – 0.03, *SE* = 0.06, *p* = 0.64).

**Table 4:** Sub-group analysis

Variable	No. of studies	SMD (95% CI)	I^2^	Q-value	df	p-value
Anxiety						
Risk of bias						
High	40	− 0.41 (− 0.57 to − 0.26)	80.73	0.64	1	0.43
Low	6	− 0.25 (− 0.62 to 0.11)	57.36			
Mode of delivery						
Mixed	1	− 0.13 (− 0.87 to 0.62)	0%	3.0	2	0.22
Onsite	33	− 0.47 (− 0.64 to − 0.30)	83.10%			
Technology	12	− 0.22 (− 0.48 to 0.04)	31.54%			
Format of delivery						
Group	28	− 0.30 (− 0.48 to − 0.11)	48.56%	2.72	2	0.26
Individual	15	− 0.53 (− 0.77 to − 0.29)	90.84%			
Mixed	1	− 0.13 (− 0.97 to 0.72)	0%			
Type of therapy						
Multicomponent	28	− 0.38 (− 0.56 to − 0.21)	80.59%	0.004	1	0.95
Relaxation only	18	− 0.39 (− 0.62 to − 0.17)	77.73%			
Booster sessions						
No	37	− 0.38 (− 0.54 to − 0.22)	70.76%	0.90	2	0.34
Yes	5	− 0.59 (− 1.00 to − 0.18)	94.96%			
Intervention focus						
Preventive	24	− 0.38 (− 0.56 to − 0.19)	74.81%	1.07	2	0.59
Treatment	21	− 0.42 (− 0.62 to − 0.22)	83.70%			
Both	1	0.25 (− 1.02 to 1.51)	0%			
Depression						
Risk of bias						
High	40	− 0.30 (− 0.55 to − 0.16)	77.92%	0.68	1	0.41
Low	10	− 0.18 (− 0.44 to 0.09)	68.35%			
Mode of delivery						
Mixed	1	− 0.23 (− 1.02 to 0.56)	0	0.15	2	0.93
Onsite	38	− 0.29 (− 0.44 to − 0.14)	70.79%			
Technology	11	− 0.24 (− 0.49 to 0.02)	57.46%			
Format of delivery						
Group	33	− 0.21 (− 0.37 to − 0.05)	70.65%	1.88	2	0.39
Individual	14	− 0.41 (− 0.65 to − 0.17)	86.06%			
Mixed	1	− 0.23 (− 1.032 to 0.57)	0%			
Type of therapy						
Multicomponent	29	− 0.26 (− 0.34 to − 0.06)	70.07%	0.14	1	0.71
Relaxation only	21	− 0.27 (− 0.45 to − 0.08)	83.02%			
Booster sessions						
No	42	− 0.31 (− 0.47 to − 0.19)	78.64%	1.75	1	0.19
Yes	4	− 0.03 (− 0.45 to 0.39)	67.58%			
Intervention focus						
Both	1	− 0.13 (− 1.36 to 1.10)	0%	0.65	2	0.72
Preventive	26	− 0.32 (− 0.49 to − 0.15)	79.92%			
Treatment	23	− 0.23 (− 0.41 to − 0.15)	74.10%			
Distress						
Risk of bias						
High	19	− 0.43 (− 0.68 to − 0.17)	86.23%	0.85	1	0.36
Low	4	− 0.71 (− 1.25 to − 0.16)	0%			
Mode of delivery						
Mixed	0	−				
Onsite	16	− 0.62 (− 0.87 to − 0.38)	88.39%	6.28	1	0.01
Technology	6	− 0.06 (− 0.42 to 0.30)	33.49%			
Format of delivery						
Group	14	− 0.47 (− 0.78 to − 0.17)	64.06%	2.31	2	0.31
Individual	6	− 0.64 (− 1.08 to − 0.19)	93.72%			
Mixed	2	0.02 (− 0.71 to 0.75)	45.24%			
Type of therapy						
Multicomponent	13	− 0.60 (− 0.90 to − 0.29)	90.07%	1.42	1	0.23
Relaxation only	10	− 0.30 (− 0.67 to 0.07)	61.69%			
Booster sessions						
No	19	− 0.38 (− 0.64 to − 0.11)	79.86%	3.28	1	0.07
Yes	4	− 0.96 (− 1.53 to − 0.39)	95.87%

**Table 5:** Meta-regression analysis showing association between anxiety, distress and depression outcome and density of dosage of interventions

Covariate	Coefficient	Standard error	Z-value	P
Anxiety				
Intercept	− 0.29	0.14	− 2.10	0.04
Number of sessions	0	0.010	− 0.40	0.69
Duration of sessions	0	0	− 1.54	0.12
Duration of programme	0.03	0.03	0.86	0.40
Distress				
Intercept	− 0.48	0.24	− 1.98	0.05
Number of sessions	0.02	0.01	1.61	0.11
Duration of sessions	0	0	− 1.46	0.14
Duration of programme	− 0.01	0.01	− 0.70	0.49
Depression				
Intercept	− 0.015	0.22	− 0.71	0.48
Number of sessions	− 0.01	0.01	− 0.93	0.35
Duration of sessions	0	0.0	− 0.66	0.51
Duration of programme	0	0.01	0.27	0.79

_Anxiety R2 analog = 44%, Distress R2 analog = 0.47, Depression R2 analog = 0.05_

The results show that active components (i.e., individual effect of each intervention component) of interventions explained 9% to 25% of variance in heterogeneity across studies targeting distress, anxiety, and/or depression. Highest variance in heterogeneity amongst different studies was explained in the interventions targeting anxiety as an outcome (25%). Overall, multicomponent interventions were associated with a better improvement in anxiety than relaxation alone, still this effect was statistically non-significant.

### Certainty of outcomes pertaining to anxiety, depression and distress using GRADE (Grading of Recommendations, Assessment, Development and Evaluations) framework

Certainty of outcomes pertaining to anxiety, distress and depression reported in the trials was assessed using the GRADE guidelines [37]. The certainty for the outcome of anxiety was downgraded by three levels to very low for serious concerns pertaining to risk of bias in the studies, substantial heterogeneity and publication bias in reporting of this outcome. The outcomes of distress and depression were downgraded to low by two levels, due to high risk of bias in intervention design and presence of substantial heterogeneity, explained by clinically heterogeneous study samples and interventions (see Additional file 2: Table S7).

## Discussion

The present systematic review and meta-analysis of 65 RCTs with 8009 adolescents is the first evidence synthesis to review the literature on the effectiveness of ‘relaxation techniques’ to reduce the symptoms of anxiety, distress and/or depression in young people aged 14–24. Majority of the studies included in our analysis were conducted in an educational setting of High-Income Countries (HICs) with adolescents with symptoms of depression and distress. The results of meta-analysis showed that relaxation techniques work across three outcomes (distress, anxiety and depression) yielding small to moderate effect sizes. Multicomponent, face-to-face delivered relaxation techniques with a booster dose were more effective.

The relaxation techniques were found to be moderately effective in reducing anxiety and distress in adolescents. This is consistent with the findings of previous reviews of relaxation techniques which yielded positive effect of relaxation techniques on reducing symptoms of anxiety in adults. In a systematic review and meta-analysis of 27 RCTs, with 1005 adults, Manzoni et al. [12] found that relaxation techniques had medium to large effect size to reduce the symptoms of anxiety in adults. Similarly, Klainin-Yobas [38] reported reduction in the symptoms of anxiety among older adults.

Our meta-analysis showed that relaxation techniques have only a small effect on improving depressive symptoms in adolescents. The finding is consistent with the findings reported in the literature where the impact of relaxation strategies to reduce the symptoms of depression is not well established [39]. Other psychological treatments like Cognitive Behavioural Therapy (CBT) are found to be more effective for the treatment of depressive symptoms, where relaxation techniques can be implemented with mild to moderate depressive symptoms as a part of multicomponent therapy [40]. A recent systematic review and network meta-analysis of school-based interventions to prevent anxiety and depression in children and adolescents [41] showed that compared with usual treatment, mindfulness and relaxation-based universal interventions were effective to reduce symptoms of anxiety in young people; however, no effect of relaxation techniques was found on depressive symptoms.

Although all included interventions varied greatly in their dose and types, overall, these interventions were more effective to reduce the symptoms of anxiety and distress and less effective to reduce symptoms of depression in adolescents. In this review, age of the participants was inversely related with reduction of anxiety symptoms. These findings are in line with what Manzoni et al. [12] reported, where relaxation techniques had more beneficial effects to treat anxiety in young people compared to older people.

The findings of this review may be of clinical importance given that there is a lack of evidence-based psychosocial interventions for anxiety and depression in adolescents, especially in low resource settings and that the relaxation techniques are simple, easy to learn, teach and use and can be delivered by health care workers across the spectrum (i.e., from non-specialist facilitators in community and primary healthcare settings to a mental health specialist). Finding of our review support the use of relaxation intervention techniques to address non-specific mental health distress according to the staged model of illness, which highlights the importance of relaxation technique as first level population mental health interventions [23, 42]. Because of their potential for scalability, effectiveness and cost-effectiveness, relaxation techniques have been recommended in the World Health Organization Mental Health Gap Action Programme (WHO mhGAP) intervention guide for the management of stress, emotional problems and depression in adolescents and have been used as an integral component of multicomponent psychological intervention packages for adolescent anxiety and depression in low resource settings [43].

### Strengths and limitations of this review

The findings of the present review should be interpreted in the context of following limitations. *Firstly*, the majority of the studies included in our analysis were conducted in educational settings of High-Income Countries (HIC), with only one study from a low- and middle-income country. Moreover, the studies included in this review had a high risk of bias, where the certainty of outcomes for depression, anxiety and distress was low. Further high-quality research, both from high- and low-income countries is needed to ascertain the evidence generated from the current review. *Secondly*, most of the included interventions were heterogeneous in their makeup and did not effectively define their individual therapeutic components (“active ingredients”). Not many of these interventions were manualized to aid in reproducibility and implementation. Moreover, we did not observe a lot of variance in number of sessions delivered across the interventions; therefore, regression analyses did not predict dose response relationship. Also, studies did not clearly state whether RTs were delivered by trained healthcare workers or untrained. Such information could provide comparative effectiveness of delivering RTs by trained versus not trained healthcare workers and might have implications on the scale-up of such intervention strategy, especially in low resource settings. Additionally, the heterogeneity in the design of the included interventions contributed to high risk of bias. This is similar to the previous systematic review and meta-analysis of relaxation techniques [12] where significant heterogeneity was observed in relaxation approaches, study participants and outcome measures. Studies show that simple and easy to follow interventions result in better quality of intervention delivery, which matters more than the “dose density” of an intervention to produce the desired clinical outcomes [44]. In the light of this evidence, we recommend that future studies should develop and test simple and easy to follow interventions for adolescents and provide detailed description of interventions using the standard intervention reporting guidelines [45].

## Conclusions

This is the first systematic review and meta-analysis of literature to evaluate the evidence on the effectiveness of relaxation techniques as an active ingredient to improve anxiety, distress and depression in adolescents. Given the potential for relaxation techniques to be scalable, effective and cost-effective in decreasing symptoms of anxiety, distress and improving depressive symptoms in adolescents; clinicians, practitioners, adolescent mental health experts, policy makers and academics may consider implementation of relaxation techniques with adolescents for potentially significant impact at-scale. However, further high-quality studies with low risk of bias, especially from low resource settings are needed to ascertain the evidence for the effectiveness of these highly used techniques.

## Supplementary Information

**Additional file 1.** Protocol_systematic_review_meta_analysis_relaxation_techniques.

**Additional file 2.** Supplementary_results.

## Data Availability

Not applicable.

## Abbreviations

RTs: Relaxation Techniques
RCTs: Randomized Controlled Trials
GRADE: Grading of Recommendations, Assessment, Development and Evaluations
SMD: Standardized Mean Difference
HIC: High Income Countries
CBT: Cognitive Behavioural Therapy
LMICs: Low- and Middle-Income Countries
PRISMA: Preferred Reporting Items for Systematic Reviews and Meta-Analyses
PICOS: Population, Intervention, Control, Outcome, Study Design
ICD: International Classification of Diseases
DSM: Diagnostic and Statistical Manual of Mental Disorders
SD: Standard Deviation
PMR: Progressive Muscle Relaxation Techniques
CBT: Cognitive Behavioural Therapy
ACT: Acceptance and Commitment Therapy
DASS: Depression, Anxiety and Stress Scale
SCAS: Spence Children’s Anxiety Scale
BAI: Beck Anxiety Inventory
BDI: Beck Depression Inventory
RADS: Reynolds’ Adolescent Depression Scale
CDACR: Child Depression Rating Scale Revised
PSWQ: Penn State Worry Questionnaire
GHQ: General Health Questionnaire
WHO mhGAP: World Health Organization Mental Health Gap Action Programme
