# Supplementary material for: Effectiveness of relaxation techniques ‘as an active ingredient of psychological interventions’ to reduce distress, anxiety and depression in adolescents: a systematic review and meta-analysis
Source: Int J Ment Health Syst. 2022 Jun 28;16:31. doi: 10.1186/s13033-022-00541-y (PMC9238062; doi:10.1186/s13033-022-00541-y)
Supplement: Supplementary file 1 — Additional file 1. Protocol_systematic_review_meta_analysis_relaxation_techniques. [file 13033_2022_541_MOESM1_ESM.docx]

**Supplementary material A**

**A systematic review and meta-analysis protocol examining the effectiveness of relaxation techniques to reduce distress, anxiety and depression in adolescents**

**Introduction:**

Depression and anxiety are the leading contributors to health burden among adolescents worldwide (Whiteford, et al., 2013). Studies conducted with adults show that most mental health problems begin between the ages of 11 and 18 years, with almost half of cases having started by the age of 14 years (Kessler et al., 2007). Given that mental health problems affect one in five people globally (Ferrari et al., 2014), there is a need for evidence-based psychological interventions for young people. Psychosocial interventions are effective in the prevention and treatment of depression and anxiety in adolescents; however, it is not fully understood which ‘ingredients’ of these interventions are more effective to prevent and treat anxiety or depression in adolescents and why.

The Wellcome Trust launched a Mental Health Program Strategy to find the next generation of treatments and approaches to transform how mental health problems of adolescents can be understood and addressed by 2030. The first commission of the mental health strategy aims to work with the mental health science community to explore and refine the ‘active ingredients- core aspects of interventions that really make a difference in preventing and treating anxiety and/or depression in young people (14-24) worldwide. The review of these active ingredients will help to answer what works for the majority of young people, in most contexts globally and why. As a part of first commission, our group will review the evidence for the effectiveness of ‘Relaxation Techniques’ for the prevention and treatment of anxiety and depression in at-risk adolescents aged 14 to 24 globally.

**Objectives**

Our primary research question is;

- How effective are relaxation techniques to reduce distress, anxiety and depression in adolescents, aged 14-24 years?

Our secondary research questions are;

- How effective are relaxation techniques to reduce adolescent distress, anxiety and depression in different settings/target populations/delivery formats?
- How many studies have used ‘relaxation technique’ as a key component of interventions to reduce distress, anxiety and depression in at-risk adolescents aged 14-24?
- How does published literature describe relaxation in the context of preventive and treatment interventions for adolescents and what techniques have been used?
- What are the settings/target populations for ‘relaxation techniques’ and in which format these techniques are delivered?

**Methods**

**Criteria for study inclusion**

**Population:** We will only consider studies focusing on adolescents (aged 14-24 years) exhibiting prodromal symptoms of depression and anxiety/distress or near cut-off scores on psychometric scales.

**Intervention:** Only those interventions will be included in which relaxation technique has been used to reduce the symptoms of distress, anxiety and depression among adolescents aged 14-24.

**Comparator condition:** All types of control arm will be included (active control group, wait list control, no intervention, treatment as usual).

**Outcomes:** Only those studies will be included which report either symptoms of distress, anxiety and/or depression as a primary or secondary outcome. We will consider both ICD/DSM criteria of diagnoses or symptom severity of anxiety and depression as measured on psychometric scales.

**Study type:** Only randomized controlled trials (with individual, cluster and step-wedge) will be included.

**Settings:** Studies from all regions will be included. All settings of interventions will be considered for inclusion.

**Search strategy for identification of studies**

Ten electronic databases will be searched, namely: PubMed, Cochrane CENTRAL, PsychInfo, Virtual Health Library, Scopus Open access, Web of Science all databases (Russian database), Psycharticles, Psychextra, Proquest Dissertation and thesis (see Table 1 for search strategy). Only English language articles will be considered for inclusion. Our search strategy includes following search terms:

**Table 1:** Search Strategy

| **Condition** | **Search terms** |
| --- | --- |
| Participants | (teenage” OR “teenagers” OR “teen” OR “teens” OR “youth” OR “young” “youngster” OR ”youngsters” OR “young adult” OR “juvenile” OR “adolescent” OR “adolescents” OR “adolescence) |
| Interventions | "psychological relaxation" OR "mental relaxation" OR "physiological relaxation" OR "therapeutic relaxation" OR "relaxation training" OR "relaxation technique*" OR breathing OR "slow breathing" OR "deep breathing" OR meditation OR "progressive muscle relaxation" OR "imagery" OR "Autogenic training" OR spirituality OR walking OR gardening OR yoga OR "T'ai chi" OR Qigong OR massage OR acupuncture OR "Feldenkrais Method" OR myotherapy OR reflexology OR self-regulation OR autosuggestion OR prayer OR hypnosis OR Pranayama OR biofeedback OR music OR art-therapy OR stress-management OR writing OR exercise OR aromatherapy OR hydrotherapy OR laughing-therapy OR food-therapy OR mindfulness) |
| Conditions | (“depression" OR “depressive disorder” OR “depressive symptoms” OR “depressed” OR “anxiety” OR “anxieties” OR “anxiety symptoms” OR “anxiety disorder” OR “anxiety disorders”) |
| Outcomes | Mental health” OR psychosocial OR “Well-being” OR “self-esteem” OR social OR suicide OR suicidality OR distress OR depress* OR stress OR anxiety OR anxious OR emotional OR internaliz* OR externaliz* |
| Study design | (“Clinical trial” OR intervention OR trial OR “randomized controlled trial” OR RCT OR “cluster randomized control trial”) |
| Region | N/A |

**Selection process**

Four reviewers working independently from one another, will review titles and abstracts, followed by full text screening of eligible studies, as per our inclusion and exclusion criteria. This process will be aided by use of Rayyan software. Discrepancies in the inclusion process will be discussed and resolved, in conjunction with senior authors.

**Data extraction**

Data extraction would be performed across three broad matrices: i) study sample ii) implementation characteristics and iii) orientation and underpinnings of intervention. Variables pertaining to study sample would include important characteristics such as age range, inclusion and exclusion criteria, and setting of intervention. Implementation characteristics would include variable such as measures of intervention fidelity, type of delivery agents and their competency assessment, supervision and training process and resource provision. While intervention will be evaluated for their theoretical underpinning using the distillation and matching framework by Chorpita et al (2005). Interventions will be categorized according to their content and then critically analyzed to identify individual active elements of interventions comprising them.

**Outcomes**

**Primary outcome:** The primary outcome is reduction in symptoms of distress, anxiety, and depression measured using self-report, psychometrically valid and reliable scales that are scored on a continuum (McElroy & Patalay, 2019). We will include data of both composite outcome as well as data reported subscale wise. We will use the study specific primary end-point (where it would be provided).

**Secondary outcomes:** The secondary outcomes will include data on i) study sample (such as age range, inclusion and exclusion criteria, and setting of intervention used ‘relaxation techniques’); ii) implementation characteristics (intervention setting, dosage and duration of relaxation techniques and type of delivery agents used) and iii) orientation and underpinnings of intervention used relaxation techniques.

**Risk of bias (quality) assessment**

Risk of bias among RCTs will be assessed using the Cochrane tool for risk of bias assessments. GRADE evidence criteria would be used to grade the certainty of evidence for these interventions for two outcomes: a) rates of anxiety disorders and their symptom severity and b) rates depressive disorders and their symptom severity. Using GRADE profiling method, the strength of evidence for these outcomes will be rated from very low to high.

**Strategy for data synthesis**

Effect size relevant quantitative data will be extracted such as mean (SD) for continuous outcomes and frequency of evens and sample size of intervention and control group for binary outcomes. A series of meta-analyses will be run, where studies will be weighted using random effects model, generating forest plots exhibiting effect size for each study along with their 95% confidence intervals. Random effects will be applied throughout the analyses due to expected clinical, methodological and statistical heterogeneity in the studies. Sensitivity analyses will be employed to assess contribution of each study especially outliers to the pooled effect size. Publication bias will be assessed using Egger’s regression statistic where there are more than ten studies. In addition, we will also visualize Begg’s funnel plot (Egger et al, 1997). Subgroup analyses will be run when specific subgroups are reported in more than 4 studies and meta-regression when covariates are reported in more than ten studies.

**Analysis of subgroups or subsets**

Subgroup analyses will be conducted based on theoretical orientation of psychological interventions, type of delivery agents, dosage density of intervention and type of population (at-risk).

**References:**

- Chorpita BF, Daleiden EL, Weisz JR. Identifying and selecting the common elements of evidence-based interventions: a distillation and matching model. Ment Health Serv Res. 2005 Mar;7(1):5-20. doi: 10.1007/s11020-005-1962-6. PMID: 15832690.
- Egger, M., Davey Smith, G., Schneider, M., & Minder, C. (1997). Bias in meta-analysis detected by a simple, graphical test. Bmj, 315(7109), 629-634. https://doi.org/10.1136/bmj.315.7109.629
- Ferrari, A. J., Norman, R. E., Freedman, G., Baxter, A. J., Pirkis, J. E., Harris, M. G., ... & Whiteford, H. A. (2014). The burden attributable to mental and substance use disorders as risk factors for suicide: findings from the Global Burden of Disease Study 2010. PloS one, 9(4), e91936.
- Kessler, R. C., Angermeyer, M., Anthony, J. C., De Graaf, R. O. N., Demyttenaere, K., Gasquet, I., ... & Kawakami, N. (2007). Lifetime prevalence and age-of-onset distributions of mental disorders in the World Health Organization's World Mental Health Survey Initiative. World psychiatry, 6(3), 168.
- McElroy E, Patalay P. In search of disorders: internalizing symptom networks in a large clinical sample. J Child Psychol Psychiatry. 2019 Aug;60(8):897-906. doi: 10.1111/jcpp.13044. Epub 2019 Mar 21. PMID: 30900257; PMCID: PMC6767473.
- Whiteford, H. A., Degenhardt, L., Rehm, J., Baxter, A. J., Ferrari, A. J., Erskine, H. E., ... & Burstein, R. (2013). Global burden of disease attributable to mental and substance use disorders: findings from the Global Burden of Disease Study 2010. The Lancet, 382(9904), 1575-1586.
