# Supplementary material for: Effectiveness of relaxation techniques ‘as an active ingredient of psychological interventions’ to reduce distress, anxiety and depression in adolescents: a systematic review and meta-analysis
Source: Int J Ment Health Syst. 2022 Jun 28;16:31. doi: 10.1186/s13033-022-00541-y (PMC9238062; doi:10.1186/s13033-022-00541-y)
Supplement: Supplementary file 2 — Additional file 2. Supplementary_results. [file 13033_2022_541_MOESM2_ESM.docx]

**Contents**

[*Figure S5: Funnel plot for anxiety outcome* 2](#_Toc106701681)

[*Figure S6: Funnel plot for distress outcome* 2](#_Toc106701682)

[*Figure S7: Funnel plot for depression outcome* 3](#_Toc106701683)

[*Figure S8: Regression of standard difference in means on duration of program for anxiety outcome* 3](#_Toc106701684)

[*Figure S9: Regression of standard difference in means on duration of session for anxiety outcome* 4](#_Toc106701685)

[*Figure S10: Regression of standard difference in means on number of sessions for anxiety outcome* 5](#_Toc106701686)

[*Figure S11: Regression of standard difference in means on duration of program for depression outcome* 6](#_Toc106701687)

[*Figure S12: Regression of standard difference in means on duration of sessions for depression outcome* 7](#_Toc106701688)

[*Figure S13: Regression of standard difference in means on number of sessions for depression outcome* 8](#_Toc106701689)

[*Figure S14: Regression of standard difference in means on duration of program for distress outcome* 9](#_Toc106701690)

[*Figure S15: Regression of standard difference in means on duration of sessions for distress outcome* 10](#_Toc106701691)

[*Figure S16: Regression of standard difference in means on number of sessions for distress outcome* 11](#_Toc106701692)

[*Table S6: Intervention characteristics (N=65)* 13](#_Toc106701693)

[*Table S7: Certainty of outcomes pertaining to anxiety, depression and distress using GRADE framework* 26](#_Toc106701694)

[*Table S8: List of outcome measures used in studies* 28](#_Toc106701695)

# *Figure S5: Funnel plot for anxiety outcome*

# *Figure S6: Funnel plot for distress outcome*

# *Figure S7: Funnel plot for depression outcome*

# *Figure S8: Regression of standard difference in means on duration of program for anxiety outcome*

# *Figure S9: Regression of standard difference in means on duration of session for anxiety outcome*

# *Figure S10: Regression of standard difference in means on number of sessions for anxiety outcome*

# *Figure S11: Regression of standard difference in means on duration of program for depression outcome*

# *Figure S12: Regression of standard difference in means on duration of sessions for depression outcome*

#

# *Figure S13: Regression of standard difference in means on number of sessions for depression outcome*

# *Figure S14: Regression of standard difference in means on duration of program for distress outcome*

# *Figure S15: Regression of standard difference in means on duration of sessions for distress outcome*

# *Figure S16: Regression of standard difference in means on number of sessions for distress outcome*

#

# *Table S6: Intervention characteristics (N=65)*

| **Sr. No.** | **Study (Author, Year)** | **Intervention name and theoretical underpinning** | **Components** | **Dosage** | **Delivery agent** | **Clinical outcome** | **Recruitment Setting** | **Beneficiary population (age [Mean (SD)], gender)** | **Effectiveness Time-points** | **Relaxation type** |
| --- | --- | --- | --- | --- | --- | --- | --- | --- | --- | --- |
|  | (Caldwell et al., 2016) | Tai chi chuan intervention | **Tai chi chuan (TCC)** (static and dynamic qigong exercises, assigning homework) | 10 weeks, 20 sessions, 60 minutes each | instructors | Anxiety | Educational settings | 18-40 (mean and SD missing)  Both | 10 weeks | Relaxation |
|  | (Gold et al., 2017) | Group Music Therapy  Theoretical basis not specified | Music, writing,  group improvisation, song contributions, song writing, playlist creation | 8 sessions | Music therapists | Depression | Educational settings | 13.84 (0.74)  Both | 3 months | Relaxation |
|  | (Robledo-Colonia et al., 2012) | Not specified | Relaxation*, Walk, Aerobic exercise, stretching, music, | Three per week, starting between week 16 and 20 of gestation and continuing for 3 months, 60 minutes each | Physiotherapist and physician | Depression | Health centre | 21 (SD missing)  Females | 3 months | Relaxation |
|  | (Harmat et al., 2008) | Music therapy  Theory: Not specified | Relaxing classical music | 3 weeks, 45 minutes | CD delivered | Depression | Home based | 22.6 (2.83)  Both | 3 weeks | Relaxation |
|  | (Nabkasorn et al., 2006) | Physical exercise/ jogging  Theory: Not specified | Physical exercise (jogging) | 8 weeks, 40 sessions, 50 minutes each | Physical fitness instructor | Depression | Educational settings | 18.8 (0.7)  Females | post training | Relaxation |
|  | (Putra et al., 2018) | None (banana consumption) | Banana consumption, walk | Missing | Self-delivered | Depression | Home based | 15-17  Females | 2 weeks | Relaxation |
|  | (MacMahon & Gross, 1988) | aerobic exercise.  not specified | Exercise, long-running, vigorous basketball | 12 weeks, 36 sessions, 40 min each. | Physical education staff | Depression | Others (Juvenile detention facilities) | 16.3 (SD missing), Male. | Post intervention | Relaxation |
|  | (Reynolds & Coats, 1986) | Relaxation training  Theoretical: Not specified | Relaxation (Assigning homework, practicing relaxation, PMR, reviewing homework) | 10 sessions, 50 minutes each | Doctoral-level school psychology graduate student ( School psychologist) | Depression and anxiety | Educational settings | 15.65 (SD missing)  Both | 5 months after the intervention | Relaxation |
|  | (Roth, 1989) | Aerobic exercise  Not specified | Aerobic exercise (Bodyguard 990 bicycle ergometer) | Only 1 sessions of 120 minutes | Not specified | Depression & Anxiety | Not specified | 20.8 (SD missing)  Both | Post intervention | Relaxation |
|  | (Roth & Holmes, 1987) | Two intervention group; aerobic exercise training and Progressive relaxation training | Aerobic Exercise Training (Running and brisk walking, stretches), Jacobson's Progressive Relaxation Training, mental imagery procedures | 11 weeks, 29 sessions, 90 minutes each | Trained instructor | Depression & Anxiety | Educational settings | 18.9 (1.3)  Both | 2 months | Relaxation |
|  | (Velasquez et al., 2015) | Yoga  Not specified | Postures (asanas), breathing exercises (pranayamas),  relaxation (yoga nidra), and meditation techniques* | 12 weeks, 24 sessions, 120 minutes each | Yoga instructor | Depression & Anxiety | Educational settings | Not specified | Post intervention | Relaxation |
|  | (Norris et al., 1992) | 3-intervention groups 1) high intensity exercise 2) moderate intensity exercise 3) stretching and flexibility  Not specified. | Aerobic exercises | 10 weeks, 20 sessions, 30 min each | Experienced instructors | Depression, anxiety and stress. | Educational settings | 16 (SD missing)  Both. | Post intervention | Relaxation |
|  | (Walsh et al., 2016) | Brief Mindfulness Training, | Mindfulness (body scan, sitting meditation, yoga) | 4 weeks, 4 sessions, 50 minutes each | Clinical psychologist | Depression | Not specified | 19.15 (SD missing)  Females | 3 weeks post | Multicomponent |
|  | (Raes et al., 2014) | Mindfulness group program  Mindfulness-Based Cognitive Therapy (MBCT) and Mindfulness-Based Stress  Reduction (MBSR) | Guided experiential mindfulness exercises (e.g., mindfulness of breathing, breathing space, body scan),  sharing of experience of these exercises; reflections in small groups, inspiring stories;  psycho-education (e.g., stress, depression, self-care), and review of homework. | 8 weeks, 8 sessions, 100 minutes each | Trained mindfulness instructor (two psychologists, one medical doctor) | Depression | Educational settings | 13- 20 (mean and SD missing)  Both | 6 months after the intervention | Multicomponent |
|  | (Sagon et al., 2018) | The Mindful Way through the Semester  Acceptance Based Behavioral Therapy (ABBT) | Mindfulness practices (awareness & acceptance, mindfulness exercises [mindfulness of breadth, inviting a difficulty]), journal  writing, social support, problem solving, and distraction or avoidance, identifying thoughts, self-awareness, behavioural choices (values concept) | 2 hours, 4 videos, 10-15 minutes | Instructor | Depression | Educational settings | 18.15 (0.46)  Both | 2 months | Multicomponent |
|  | (Stasiak et al., 2014) | Computer-Based CBT & Computerized Psychoeducation  CBT & psychoeducation | cCBT (Behavioural activation and pleasant activity scheduling, Problem solving, conflict resolution, Cognitive restructuring: identifying unhelpful thoughts, challenging unhelpful thoughts, thought stopping. Relaxation techniques) computerized psychoeducation i.e. CPE [Depression education and mind-body connection, Physical health (nutrition, exercise, sleep) and how to stay healthy, Friendships, conflict and anger, Time management: making schedules and routines (exam time), reaching goals (setting realistic goals and rewards)], Stress management: signs of stress, some stress can be OK but how much is too much?, Personal fulfilment (creativity, music, poetry, community involvement)] (imploding and exploding) |  | Online Modules | Depression | Online | 13-18 (mean and SD missing)  Both | 1 month | Multicomponent |
|  | (Cui et al., 2016) | Group Cognitive Behavioural Intervention having relaxation component  CBT | Weekly goal setting, relaxation techniques, homework task, psychoeducation about depression; 2) behavioural activation,  graded exposure, (identifying thoughts and feelings);  problem solving, identifying affect, identifying thoughts, cognitive restructuring, distraction, mood monitoring, assigning homework | 8 weeks, 8 sessions, 120 minutes | Senior graduate student | Depression | Educational settings | 19.42 (1.66) Both | post intervention | Multicomponent |
|  | (Felver et al., 2015) | Yoga  Not specified | Yoga (physical exercises, muscular strength, balance, coordination, and somatic awareness, breathing exercises), mindfulness practices (ability to control attention, mind-body awareness), somatic self-awareness, introducing basic physiological response of stress (i.e., the fight-or-flight response), mindful breathing, brief relaxation activity (e.g.,  5 min spent lying on back and focusing on somatic  sensations of non-activity), group discussion (e.g., dealing with stress, non-judgmental self-awareness). | 3 weeks, 15 sessions, 35 minutes each | Yoga practitioner | Depression | Educational settings | 15 (SD missing)  Both | Post intervention | Multicomponent |
|  | (Khalsa et al., 2012) | Yoga intervention  Theoretical basis not specified | Simple yoga postures, breathing exercises, visualization, and games with an emphasis on fun, relaxation (breathing exercises), mindfulness, meditation, self-awareness, non-violence, mind–body interactions and awareness, body systems, stress management, emotional intelligence, self-talk and critical voice, contentment, discipline, decision making, values and principles, commitment, and acceptance | 11 weeks, 23 -32 sessions, 30-40 minutes | Yoga instructors | Stress | Educational settings | 16.8 (0.6)  Both | immediate | Multicomponent |
|  | (de Vibe et al., 2013) | Mindfulness-Based Stress Reduction (MBSR) programme  Mindfulness | MBSR programme (physical and mental exercises, didactic teaching on mindfulness, stress management and mindful communication using a course manual and CDs for home practice, a group process to facilitate reflections) | 7 weeks, 7 sessions, duration of 1-6 sessions was 1.5 hours, while 7th session was of 6 hours, 30 minutes’ homework practice | Trained MBSR instructors | Stress | Educational setting | 23 (SD missing) both | 2 weeks | Multicomponent |
|  | (Erogul et al., 2014) | MBSR programme  Mindfulness | Mindfulness (meditation, body scan, and breathing-based yoga), psychoeducation (mindfulness, provide a cognitive curriculum about understanding stress and how best to manage reactivity) | 8 weeks, 8 sessions, 75 minutes each. | Psychotherapist | stress | Educational settings | 23.5 (SD missing) Both. | 8 weeks | Multicomponent |
|  | (Phang et al., 2015) | Mindful-Gym: mindfulness-based stress management (MBSM/Mindful- Gym) program  Mindfulness | Mindfulness (e.g. mindful breathing, mindful stretching, and body scan). | 5 weeks, 5 sessions, 120 minutes each. | Psychiatrist | stress | Educational settings | Experimental group: 21.14 (SD = 1.10), control group : 20.94 (SD = 1.17), Both | 1 week | Multicomponent |
|  | (Scholten et al., 2016) | Dojo (Game)  Theoretical basis (Not specified) | Biofeedback system, guided imagery, positive self-talk, muscle relaxation, deep breathing | 3 weeks, 6 sessions, 60-120 minutes each | Game | Anxiety | Educational settings | 11-15 9 (mean SD missing)  Both | 3 weeks | Multicomponent |
|  | (Grassi et al., 2011) | A Stress Inoculation Training-based protocol  CBT | Stress management (Imaginary exercises, emotional regulation, muscular relaxation, relaxation exercise) | 1 week, 6 sessions | Multimedia (CD, DVD, Mp3, Universal mobile telecommunication system) | Anxiety | Educational settings | 20.86 (1.27)  Females | Pre post | Multicomponent |
|  | (Dvořáková et al., 2017) | Learning to BREATHE (L2B) program  Mindfulness-based curriculum | Breathing, Stress management, mindfulness (three mindful breaths), emotional regulation, guided meditations (body scan, loving-kindness practice) | 8 weeks, 8 sessions, each session of 80 minutes | Not specified | Depression and anxiety | Others | 18.2 (0.4)  Both | 6 weeks | Multicomponent |
|  | (Vázquez et al., 2012) | Relaxation training | Breathing, meditation, PMR, Imagery, mood monitoring, Assigning homework, | 8 sessions, 90 minutes each | Clinical psychology PhD students | Depression and anxiety | Not specified | 23.3 (SD missing)  Both | 3 months | Multicomponent |
|  | (McGrady et al., 2012) | Wellness program  Mindfulness based stress reduction | Stress Management (deep breathing, progressive relaxation, guided imagery, survival thinking, mindfulness meditation, nutrition, coping, managing fatigue and anxiety and balancing life), relaxation technique (mindful breathing or imagery) | 8 sessions, 45 minutes each | Experienced practitioner (psychologist, counselor, or physician) | Depression and anxiety | Others | First year medical students (mean, SD missing)  Both | 9 months follow up | Multicomponent |
|  | (Levin et al., 2017) | Acceptance and commitment therapy  ACT with mindfulness components | Acceptance, values, mindfulness (audio guided mindfulness exercises* mindful breathing exercise; Mindfulness of internal experiences), goal setting, ACT skills, writing. | 3 weeks, 6 sessions | Online | Depression and anxiety | Educational settings | 21.61 (5.48)  Both | After completion of sessions: 4 weeks | Multicomponent |
|  | (Blake et al., 2018) | Sleep SENSE intervention  Cognitive behavioral Therapy | Stress management, Psychoeducation,  sleep education, sleep hygiene, stimulus control, and cognitive restructuring, Motivational interviewing techniques, problem solving, identifying thoughts, sleep diaries, thought monitoring, assigning & reviewing homework, goal setting, empathy. | 7 sessions, each session of 90 minutes | Clinical psychologists or graduate clinical psychologists | Depression and anxiety | Educational settings | 14.48 (SD missing)  Both | 7 weeks | Multicomponent |
|  | (Merry et al., 2012) | Computerised CBT (SPARX)  CBT | CBT (Psychoeducation about depression, Relaxation: controlled breathing, Activity scheduling and behavioural activation, progressive muscle relaxation, Basic communication, and interpersonal skills, dealing with strong emotions, Interpersonal skills, Problem solving, Cognitive restructuring, recognizing different types of negative automatic thoughts, Mindfulness: tolerating distress) | 7 modules | CD delivered | Depression and anxiety | Online | 15.6 (SD missing)  Both | 3 months follow up | Multicomponent |
|  | (Seligman et al., 2007) | Classroom-based cognitive-behavioral workshop  CBT with relaxation | identifying automatic negative thoughts and underlying beliefs, replacing automatic negative thoughts, thought stopping, distraction, behavioural activation, problem solving, assertiveness, stress management  (relaxation training), emotional regulation | 8 weeks, 8 sessions, 120 minutes each | Trained and experienced cognitive therapists | Depression and anxiety | Educational settings | First year undergraduates (mean age, SD missing  Both | 6 months | Multicomponent |
|  | (Kenardy et al., 2003) | Online Anxiety Prevention Program  CBT with relaxation | Psychoeducation about anxiety, relaxation training,  interceptive exposure, cognitive restructuring and relapse prevention, self-monitoring, | 12 weeks 6 sessions | Online | Depression and anxiety | Both educational settings & home | 19.92 (4.78)  Both | 6 weeks: Post session | Multicomponent |
|  | (Seligman et al., 2000) | Cognitive–behavioural workshop  CBT | Identifying automatic negative thoughts and underlying beliefs; replacing automatic negative thoughts, stopping, distraction techniques); behavioural activation strategies, anti-procrastination techniques, creative problem solving, assertiveness training, interpersonal skills (active listening, taking each other’s perspectives, controlling emotions, passive vs. assertive vs. aggressive behaviours), stress management (relaxation training*), games, emotional regulation, role play, assigning & reviewing homework, motivational interviewing, | 8 sessions, 120 minutes each | Cognitive therapist | Depression and anxiety | Educational settings | 1st year Under-graduates (mean, SD missing)  Both | 8 week | Multicomponent |
|  | (Calear et al., 2009) | MoodGYM Program  Theory: CBT | CBT (identify negative thinking patterns and change them, identify the situations or events that may precipitate negative thinking, Relaxation techniques, Teaches users about relationship breakups and how to cope with them, problem solving) | 5 weeks, 5 sessions, 20-40 minutes | Online/self-paced | Depression and anxiety | Educational settings | 14.34 (SD missing)  Both | post intervention | Multicomponent |
|  | (Chen et al., 2013) | Mindfulness meditation training  Mindfulness | Mindfulness (cognitive practice of mindfulness meditation, concentration on breathing with eyes closed, non-judgmental awareness of thoughts, feelings, and sensations with a focus on the flow of breath through the nostrils; body scan, paying attention to the breath and focusing on the dynamic sensations of the whole body, mindfulness with the help of background music) | 1 week, 7 sessions, 30 minutes | Senior psychological counsellor | Anxiety and Depression | not specified | 19.5 (0.87)  Both | post intervention | Multicomponent |
|  | (Delgado et al., 2010) | Mindfulness based stress reduction  Mindfulness | Mindfulness (guided meditation, breathing, re-cognition of the experience of the present mental state.; body scan, focus on present mental and emotional state, generating positive, feelings of acceptance; relaxation training (guided relaxation practice, muscle relaxation, relaxation training of speech and imagination) | 5 weeks, 10 sessions, 60 minutes | Not specified | Anxiety and Depression | not specified | 18-24 (mean SD missing)  Females | post intervention | Multicomponent |
|  | (Shapiro et al., 1998) | Meditation based stress reduction and relaxation  Mindfulness | Body scan, sitting meditation, hatha yoga, mindful breathing, lovingkindness and forgiveness meditation, empathy, mindful listening skills | 8 weeks, 7 sessions, 150 minutes | Not specified | Anxiety and Depression | Educational setting | Age is not specified, Both | Post intervention | Multicomponent |
|  | (Astin, 1997) | Mindfulness meditation- based stress reduction  Mindfulness and CBT based | Body scan, sitting meditation, hatha yoga | 8 weeks, 8 sessions, 120 minutes | Researcher trained in meditations | Anxiety and Depression | Both educational and residential setting | Age is not specified, Both | 6 to 9 months | Multicomponent |
|  | (Shearer et al., 2015) | Mindfulness meditation intervention  mindfulness | Mindfulness (breathing exercises, basic yoga including light stretching and  balancing exercises, short meditation sessions, and education, about the physiology of the stress response.) | 4 weeks, 4 sessions, 60 mins. | Not specified | Depression, anxiety and stress. | Educational settings | Undergraduate students, age is not specified, Both. | Post intervention | Multicomponent |
|  | (Hilyer et al., 1982) | Physical fitness program & counselling | Flexibility exercise, praise, reinforcements, movements | 20 weeks, 60 sessions, 90 minutes each. | Physical fitness trainer | Depression and anxiety | Educational setting | Experimental group: 17.01 (SD missing), control group: 16.90 (SD missing), Male. | Post intervention | Multicomponent |
|  | (Melnyk et al., 2009) | The COPE Healthy Lifestyles TEEN Program  CBT | Psychoeducation (creating a healthy lifestyle, strategies to build self-esteem, stress management, goal setting, effective communication, nutrition, and physical activity), physical activity (Frisbee, kickball, walking, and relay games), self-esteem, positive self-talk, goal setting, problem solving, stress & coping, emotional & behavioural regulation, communication skills, stretching, food groups, nutrients, influence of feeling on eating, social eating strategies, role plays | 9 weeks, 15 sessions, 50 minutes each | Research personnel | Depression & Anxiety | Educational settings | 14-16 (mean SD missing)  Both | Post intervention | Multicomponent |
|  | (Ștefan et al., 2018) | Mindfulness-Based Stress Reduction Intervention  Mindfulness-based stress reduction   and mindfulness-based cognitive therapy | Psychoeducation (mindfulness, initial meditation and body scan)  Meditation, walk, mindfulness (sound and walking, breathing with spaciousness) identifying thoughts, | 6 weeks, 6 sessions, 90-120 minutes each session | CBT trainer with experience in mindfulness meditation | Anxiety and stress | Not specified | 18.92 (1.04)  Females | 1 week | Multicomponent |
|  | (Chiauzzi et al., 2008) | Mystudentbody-stress website  CBT | Stress management (Strategies include tips on time management, developing good sleep habits, practicing relaxation skills or meditation, handling depression and anxiety, developing social support, and communicating with family members) | 4 sessions, 20 minutes each | Self-delivered | Anxiety and stress | Not specified | 18-24 (mean SD missing)  Both | 6 months | Multicomponent |
|  | (Saravanan & Kingston, 2014) | PMRT, systematic desensitization  Theoretical basis (Not specified) | Psychoeducation*, PMR (assigning homework), systematic desensitization (exposure, relaxation technique) | 3 weeks, 5 sessions | Clinical psychologist | Anxiety and stress | Educational settings | 19 (1.04)  Both | 3 weeks | Multicomponent |
|  | (Fleming et al., 2012) | Computerized CBT, SPARX  CBT | Psychoeducation*, problem solving, relaxation*, social skills, cognitive restructuring, activity scheduling | 5 weeks, 7 sessions, 30 minutes each | Self/ computerized | Anxiety and stress | Educational settings | 14.9 (0.79)  Males | 5 weeks | Multicomponent |
|  | (Deckro et al., 2002) | Mind/Body Intervention  CBT and relaxation | Relaxation (Diaphragmatic breathing, Guided imagery, Progressive muscle relaxation, Brief relaxation exercises (“minis”), yoga stretches, mindfulness.),  Cognitive behavioural interventions (Identifying automatic thoughts, Challenging cognitive distortions, Affirmations, Goal setting)  Psychoeducation (Stress, stress symptoms, and coping, Mind/body connection, Physiology of stress and the relaxation response, Weekly discussion of relaxation practice) | 6 weeks, 6 sessions, 90 minutes each | Trainers | Anxiety and stress | Educational settings | 24 (SD missing)  Both | immediate | Multicomponent |
|  | (Nguyen-Feng et al., 2017) | Web-based stress management intervention  Theory: Not specified | Present control+mindfulness (psychoeducational video on mindfulness, completed mindfulness exercises such as listening to mindfulness meditation audio files that taught breath awareness and noticing emotions that arise) | 4 weeks, 3 sessions, 20 minutes each | Not specified | Anxiety and stress | Online | Age is not specified  Both | 4 weeks | Multicomponent |
|  | (Zhang et al., 2018) | Mindfulness-based Tai Chi Chuan | Breathing, meditation, Tai-chi, mindfulness (mindfulness based breathing, walking meditation), muscle relaxation, gentle stretches, chest expanding, knee movement, shape up exercise | 16-8 weeks, twice a week, 90 minutes each | Experienced Physical Exercise coaches | Depression and stress | Educational settings | 18.41 (2.01)  Both | 8 weeks | Multicomponent |
|  | (Bluth et al., 2016) | Learning to BREATHE (L2B)  Mindfulness based stress reduction | Meditation, walk, yoga, music, exercise, mindfulness  (the body scan, sitting, meditation, lovingkindness practice, walking meditation and mindful movement, mindfulness of thoughts and emotions) | 11 sessions, 50 minutes each | Experienced mindfulness practitioner | Depression, anxiety and stress | Educational settings | 16.8 (1.3)  Both | post-intervention | Multicomponent |
|  | (Flett et al., 2019) | Two Mobile mindfulness meditation applications (Headspace & Smiling Mind)  Mindfulness | Mindfulness (mindful breathing, body scan, mindful eating, sitting meditation, other guided meditations) | 4 weeks, 10 sessions, 10 minutes each | Online | Depression, anxiety, and stress | Others | 20.08 (2.8)  Not specified | 10 days | Multicomponent |
|  | (Song & Lindquist, 2015) | Mindfulness-based stress reduction (MBSR)  Mindfulness | Mindfulness (hatha yoga, sitting, eating & walking meditation, gentle stretching, breath-work, body scan, and, assigning & reviewing homework) | 8 weeks, 8 sessions, 120 minutes each | Trained instructor | Depression, anxiety, and stress | Educational settings | 19.6 (1.7)  Both | Post intervention (8 weeks) | Multicomponent |
|  | (Rentala et al., 2019) | Stress management program  Chan’s body–mind–spirit (BMS) model & psychoeducation | Psychoeducation strategies (emotional management, stress reduction techniques such as acupressure exercises, breathing techniques and meditation, connecting to spiritual and self‑healing resources),  holistic stress management program (singing activity hand swinging exercises, breathing exercises, clay therapy, meditation acupressure exercises craftwork stress sorting exercise focusing on stressful situation, their reaction, and ways of coping, therapeutic writing drawing, self-love techniques mirror exercises, progressive muscle relaxation storytelling, mindful eating) | 4 weeks, 8 session, 90-120 minutes | Psychiatrist | Depression, anxiety and stress | Educational settings | 16-19 (SD missing)  Females | 1 month | Multicomponent |
|  | (Hall et al., 2018) | Low intensity Health enhanced mindfulness intervention  Mindfulness | Mindfulness & meditation (mindful eating, sitting, and breathing), mindfulness exercises (body scan and mindful walking), home based practice | 7 weeks, 7 sessions, 90 minutes each | Facilitator head of student counselling section | Depression, anxiety, and stress | Home based | 22.30 (2.63)  Both | week 7 | Multicomponent |
|  | (Hindman et al., 2014) | Mindful Stress Management (MSM)  MBSR, mindfulness-based cognitive therapy (MBCT and ACT) | Psychoeducation (mindfulness), stress management [mindfulness (a focus on the present  moment, acceptance, reviewing progress, mindful walking, eating,  listening to music, meditation, free association task] | 6 weeks, 6 sessions, 60 minutes each | Group leaders. One co-leader (experienced clinical psychologist having mindfulness (meditation) experiences) | Depression, anxiety, and stress | Educational settings | 22.35 (SD missing)  Both | immediate | Multicomponent |
|  | (Levin et al., 2019) | Acceptance and commitment therapy & Mental Health Education Website  ACT with mindfulness component | Costs of experiential avoidance, (diffusion), (mindfulness), (acceptance of difficult emotions), (clarifying personal values), and (committed action and goal setting), breathing mindful exercise, imagery, goal setting. | 3 weeks, 2 sessions | Online | Depression, anxiety, and stress | Educational settings | 20.51 (2.73)  Both | 3 month | Multicomponent |
|  | (Ellis et al., 2011) | MoodGYM (online CBT) and Mood Garden (online social support)  CBT | Problem solving, enhancing relationship, stress management (relaxation), identifying thoughts | 3 weeks, 5 sessions, 60 minutes | Online | Depression, anxiety, and stress | Others | 19.67 (1.66)  Both | Post intervention | Multicomponent |
|  | (Gallego et al., 2015) | mindfulness training  Mindfulness Based Cognitive Therapy (MBCT) | Body scan, mindful breathing, breathing space, yoga, sitting meditation | 8 weeks, 8 sessions, 60 minutes | Therapist | Anxiety, Depression and Stress | both educational and home based | 20.07 (SD missing)  Both | Post intervention | Multicomponent |
|  | (Berger et al., 1988) | Jogging, Benson relaxation  Not specified | Jogging, Benson relaxation, group interaction | 12 weeks, 14 sessions, 45 minutes each | Not specified | Depression, Anxiety & stress | Not specified | 20 (SD missing)  Both | Post intervention | multicomponent |
|  | (Van Aubel et al., 2020) | Acceptance and Commitment Therapy in Daily-Life (ACT-DL)  Behavioural therapy | ACT+mindfulness awareness, exercise | 5 sessions, 90 minutes each | Trained therapist | Depression, Anxiety & stress | community | 21.36 (2.39)  Both | Post intervention, 6 months, 12 months | Multicomponent |
|  | (Nguyen-Feng et al., 2016) | Mindfulness | Stress reduction videos, mindfulness exercise-meditation | 4 weeks, 3 sessions, 20 minutes | Not specified | Depression and Anxiety | college | 18-21 (SD missing)  Both | Post intervention, 3 weeks | Multicomponent |
|  | (Warnecke et al., 2011) | Guided mindfulness practice | Mindfulness | 8 weeks, 30 minutes | audio compact disc  (CD) | Depression, anxiety and stress | university | 23.92 (3.2)  Both | Baseline, 8 weeks, 16 weeks | multicomponent |
|  | (Moir et al., 2016) | Peer-Support and Mindfulness Program | Mindfulness | 34 weeks,18 sessions, | peer leaders | Depression and Anxiety | university | 21 (SD missing)  Both | Post intervention, 6 months | multicomponent |
|  | (Nguyen-Feng et al., 2019) | Mindfulness | Psychoeducation, written exercise, motivational interviewing techniques, self-monitor | 4 weeks | online course management system | Depression and Anxiety | college | 21.3 (SD missing)  Both | Post intervention, and 2  follow-ups (2–3 weeks and 4 –5 weeks post intervention) | Multicomponent |
|  | (Levin et al., 2016) | Acceptance and commitment therapy & Mental Health Education Website | Defining values (e.g., values as a direction animation); clarifying personal values (e.g., card sort); reflecting on values (e.g., journaling); defining SMART and values-based goals; setting a values-based goal for the week; summary of session, Reflect on actions consistent with values (day 3 text); reflect on important value and a goal for the day (day 6 text), Reminders to work on session 1 goal and to practice reflecting on values you admire in others (day 2 e-mail) or on values engaged in that day (day 4 e-mail), Intro and definition of mindfulness; mindful breathing exercise; instructions to practice mindful breathing, Review of session 1; identifying internal barriers; problems with control strategies (e.g., passengers on the bus animation); defining willingness (e.g., and vs. but exercise); practicing willingness (e.g., breath holding exercise); setting a willingness goal for the week; summary of session, Prompts to practice willingness, Reminders to work on session 2 goal and to practice the NAME acceptance technique (day 2 e-mail) or future ways to practice ACT skills, Mindfulness of internal experiences, labelling mindfulness exercise, resources for practicing other exercises | 3 weeks | online | Depression and Anxiety | university | 20.51(2.73)  Both | Post intervention | Multicomponent |
|  | (Hazlett-Stevens & Oren, 2017) | Self-help bibliotherapy format of the evidence-based mindfulness-based stress reduction (MBSR) intervention | Intervention was basically a book on Mindfulness-Based Stress Reduction. It was comprised of 10 chapters. It was comprised of formal and informal mindfulness practices, and mp3 recordings of guided practice instructions included with the workbook. Participants were required to do reflection exercises as well | 10 sessions,10 weeks | Self help | Depression and Anxiety | college | 22.1 (4.7)  Both | Post intervention | Multicomponent |

**_Abbreviations:_** _ACT, Acceptance and Commitment Therapy; CBT, Cognitive Behavioral Therapy; MBSR, Mindfulness-Based Stress Reduction; PMR, progressive Muscle Relaxation_

# *Table S7: Certainty of outcomes pertaining to anxiety, depression and distress using GRADE framework*

| **Certainty assessment** | | | | | | | **№ of patients** | | **Effect** | | **Certainty** | **Importance** |
| --- | --- | --- | --- | --- | --- | --- | --- | --- | --- | --- | --- | --- |
| **№ of studies** | **Study design** | **Risk of bias** | **Inconsistency** | **Indirectness** | **Imprecision** | **Other considerations** | **relaxation training** | **treatment as usual** | **Relative (95% CI)** | **Absolute (95% CI)** |  |  |
| **Anxiety (assessed with: Psychometric scales)** | | | | | | | | | | | | |
| 46 | Randomized trials | serious ^a^ | serious ^b^ | not serious | not serious | publication bias strongly suspected ^c^ | 2486 | 2759 | - | SMD **0.39 SD lower** (0.52 lower to 0.25 lower) | ⨁◯◯◯ VERY LOW | CRITICAL |
| **Depression (assessed with: Psychometric scales)** | | | | | | | | | | | | |
| 50 | Randomized trials | serious ^d^ | serious ^e^ | not serious | not serious | none | 2719 | 3013 | - | SMD **0.28 SD lower** (0.4 lower to 0.15 lower) | ⨁⨁◯◯ LOW | CRITICAL |
| **Distress (assessed with: Psychometric instruments)** | | | | | | | | | | | | |
| 23  **CI:** Confidence interval; **SMD:** Standardized mean difference Explanations a. A total of 40 out of 46 studies were rated as having a high risk of bias overall, with >= matrices rated as high risk on Cochrane tool for risk of bias assessment in RCTs. Subgroup analyses using mixed effects revealed high risk studies yielding higher effect sizes than studies at low risk of bias. However, this subgroup difference was statistically non-significant.  b. The reporting of this outcome was substantially heterogeneous with an I squared of 79%, this was explained by clinically heterogeneous population and interventions.  c. Egger's regression test revealed significant publication bias (P=0.02)  d. A total of 40 out of 50 studies were rated as having a high risk of bias overall, with >= matrices rated as high risk on Cochrane tool for risk of bias assessment in RCTs. Subgroup analyses using mixed effects revealed high risk studies yielding higher effect sizes than studies at low risk of bias. However, this subgroup difference was statistically non-significant.  e. The reporting of this outcome was substantially heterogeneous with an I squared of 73%, this was explained by clinically heterogeneous population and interventions.  f. 19 out 23 studies were rated as having high risk of bias, with studies with low risk of bias yielding lower effect sizes.  g. The reporting of this outcome was substantially heterogeneous with an I squared of 85%, this was explained by clinically heterogeneous population and interventions. | Randomized trials | serious ^f^ | serious ^g^ | not serious | not serious | none | 1122 | 1124 | - | SMD **0.48 SD lower** (0.71 lower to 0.24 lower) | ⨁⨁◯◯ LOW | CRITICAL |

# *Table S8: List of outcome measures used in studies*

| **Depression**   - - Beck depression inventory (BDI-II)   - Centre for Epidemiologic Studies Depression Scale (CES-D)   - The Profile of Mood States (POMS)   - Reynolds’ Adolescent Depression Scale (RADS)   - Patient Health Questionnaire 9 (PHQ-9)   - Child Depression Rating Scale Revised (CDACR)   - Montgomery Åsberg Depression Rating Scale (MADRS)   - Self-Rating Depression Scale (SDS)   - Inventory of Depressive Symptomatology, Self-Report (IDS-SR)   - Zung Self-Rating Depression Scale (SDS)   - Kessler 10 depression scale (K10)   - Brunel University Mood Scale (BRUMS) |
| --- |
| **Depression/anxiety**   - - Depression, Anxiety and Stress Scale (DASS) |
| **Anxiety**   - - The Spielberger State-Trait Anxiety Inventory (STAI)   - Zung Self-Rating Anxiety Scale (SAS)   - Beck anxiety inventory (BAI)   - State Trait anxiety (STAI)   - The Anxiety Sensitivity Index   - Spence Children’s Anxiety Scale (SCAS)   - Hospital Anxiety and Depression Scale–Anxiety Subscale (HADS-A)   - Revised Children’s Manifest Anxiety Scale (RCMAS) |
| **Distress**   - - Perceived Stress Scale   - Kessler perceived distress scale   - Generalized Anxiety Disorders (GAD) scale   - The Chinese version of the Perceived Stress Scale (CPSS)   - Educational Stress Scale for Adolescents |

References

Astin, J. A. (1997). Stress reduction through mindfulness meditation. Effects on psychological symptomatology, sense of control, and spiritual experiences. *Psychother Psychosom*, *66*(2), 97-106. <https://doi.org/10.1159/000289116>

Berger, B., Friedmann, E., & Eaton, M. (1988). Comparison of Jogging, the Relaxation Response, and Group Interaction for Stress Reduction. *Journal of Sport & Exercise Psychology*, *10*, 431-447. <https://doi.org/10.1123/jsep.10.4.431>

Blake, M. J., Blake, L. M., Schwartz, O., Raniti, M., Waloszek, J. M., Murray, G., Simmons, J. G., Landau, E., Dahl, R. E., McMakin, D. L., Dudgeon, P., Trinder, J., & Allen, N. B. (2018). Who benefits from adolescent sleep interventions? Moderators of treatment efficacy in a randomized controlled trial of a cognitive-behavioral and mindfulness-based group sleep intervention for at-risk adolescents. *J Child Psychol Psychiatry*, *59*(6), 637-649. <https://doi.org/10.1111/jcpp.12842>

Bluth, K., Campo, R. A., Pruteanu-Malinici, S., Reams, A., Mullarkey, M., & Broderick, P. C. (2016). A School-Based Mindfulness Pilot Study for Ethnically Diverse At-Risk Adolescents. *Mindfulness (N Y)*, *7*(1), 90-104. <https://doi.org/10.1007/s12671-014-0376-1>

Caldwell, K., Bergman, S., Collier, S., Triplett, N., Quin, R., Bergquist, J., & Pieper, C. (2016). Effects of tai chi chuan on anxiety and sleep quality in young adults: Lessons from a randomized controlled feasibility study. *Nature and Science of Sleep*, *Volume 8*, 305-314. <https://doi.org/10.2147/NSS.S117392>

Calear, A. L., Christensen, H., Mackinnon, A., Griffiths, K. M., & O'Kearney, R. (2009). The YouthMood Project: a cluster randomized controlled trial of an online cognitive behavioral program with adolescents. *J Consult Clin Psychol*, *77*(6), 1021-1032. <https://doi.org/10.1037/a0017391>

Chen, Y., Yang, X., Wang, L., & Zhang, X. (2013). A randomized controlled trial of the effects of brief mindfulness meditation on anxiety symptoms and systolic blood pressure in Chinese nursing students. *Nurse Educ Today*, *33*(10), 1166-1172. <https://doi.org/10.1016/j.nedt.2012.11.014>

Chiauzzi, E., Brevard, J., Thum, C., Decembrele, S., & Lord, S. (2008). MyStudentBody-Stress: an online stress management intervention for college students. *J Health Commun*, *13*(6), 555-572. <https://doi.org/10.1080/10810730802281668>

Cui, L., He, F., Han, Z., Yang, R., Xiao, J., & Oei, T. P. (2016). A Brief Group Cognitive-Behavioral Program for the Prevention of Depressive Symptoms in Chinese College Students. *International Journal of Group Psychotherapy*, *66*, 291-307. <https://doi.org/10.1080/00207284.2015.1111098>

de Vibe, M., Solhaug, I., Tyssen, R., Friborg, O., Rosenvinge, J. H., Sørlie, T., & Bjørndal, A. (2013). Mindfulness training for stress management: a randomised controlled study of medical and psychology students. *BMC Med Educ*, *13*, 107. <https://doi.org/10.1186/1472-6920-13-107>

Deckro, G. R., Ballinger, K. M., Hoyt, M., Wilcher, M., Dusek, J., Myers, P., Greenberg, B., Rosenthal, D. S., & Benson, H. (2002). The evaluation of a mind/body intervention to reduce psychological distress and perceived stress in college students. *J Am Coll Health*, *50*(6), 281-287. <https://doi.org/10.1080/07448480209603446>

Delgado, L. C., Guerra, P., Perakakis, P., Vera, M. N., Reyes del Paso, G., & Vila, J. (2010). Treating chronic worry: Psychological and physiological effects of a training programme based on mindfulness. *Behav Res Ther*, *48*(9), 873-882. <https://doi.org/10.1016/j.brat.2010.05.012>

Dvořáková, K., Kishida, M., Li, J., Elavsky, S., Broderick, P. C., Agrusti, M. R., & Greenberg, M. T. (2017). Promoting healthy transition to college through mindfulness training with first-year college students: Pilot randomized controlled trial. *J Am Coll Health*, *65*(4), 259-267. <https://doi.org/10.1080/07448481.2017.1278605>

Ellis, L., Campbell, A., Sethi, S., & O'Dea, B. (2011). Comparative randomized trial of an online cognitive-behavioral therapy program and an online support group for depression and anxiety. *Journal of CyberTherapy and Rehabilitation*, *4*, 461-467.

Erogul, M., Singer, G., McIntyre, T., & Stefanov, D. G. (2014). Abridged mindfulness intervention to support wellness in first-year medical students. *Teach Learn Med*, *26*(4), 350-356. <https://doi.org/10.1080/10401334.2014.945025>

Felver, J. C., Butzer, B., Olson, K. J., Smith, I. M., & Khalsa, S. B. (2015). Yoga in public school improves adolescent mood and affect. *Contemp Sch Psychol*, *19*(3), 184-192. <https://doi.org/10.1007/s40688-014-0031-9>

Fleming, T., Dixon, R., Frampton, C., & Merry, S. (2012). A pragmatic randomized controlled trial of computerized CBT (SPARX) for symptoms of depression among adolescents excluded from mainstream education. *Behav Cogn Psychother*, *40*(5), 529-541. <https://doi.org/10.1017/s1352465811000695>

Flett, J. A. M., Hayne, H., Riordan, B. C., Thompson, L. M., & Conner, T. S. (2019). Mobile Mindfulness Meditation: a Randomised Controlled Trial of the Effect of Two Popular Apps on Mental Health. *Mindfulness*, *10*(5), 863-876. <https://doi.org/10.1007/s12671-018-1050-9>

Gallego, J., Aguilar-Parra, J. M., Cangas, A. J., Langer Á, I., & Mañas, I. (2015). Effect of a mindfulness program on stress, anxiety and depression in university students. *Span J Psychol*, *17*, E109. <https://doi.org/10.1017/sjp.2014.102>

Gold, C., Saarikallio, S., Crooke, A. H. D., & McFerran, K. S. (2017). Group Music Therapy as a Preventive Intervention for Young People at Risk: Cluster-Randomized Trial. *J Music Ther*, *54*(2), 133-160. <https://doi.org/10.1093/jmt/thx002>

Grassi, A., Gaggioli, A., & Riva, G. (2011). New technologies to manage exam anxiety. *Stud Health Technol Inform*, *167*, 57-62.

Hall, B. J., Xiong, P., Guo, X., Sou, E. K. L., Chou, U. I., & Shen, Z. (2018). An evaluation of a low intensity mHealth enhanced mindfulness intervention for Chinese university students: A randomized controlled trial. *Psychiatry Res*, *270*, 394-403. <https://doi.org/10.1016/j.psychres.2018.09.060>

Harmat, L., Takács, J., & Bódizs, R. (2008). Music improves sleep quality in students. *J Adv Nurs*, *62*(3), 327-335. <https://doi.org/10.1111/j.1365-2648.2008.04602.x>

Hazlett-Stevens, H., & Oren, Y. (2017). Effectiveness of Mindfulness-Based Stress Reduction Bibliotherapy: A Preliminary Randomized Controlled Trial. *J Clin Psychol*, *73*(6), 626-637. <https://doi.org/10.1002/jclp.22370>

Hilyer, J. C., Wilson, D. G., Dillon, C., Caro, L., Jenkins, C., Spencer, W. A., Meadows, M. E., & Booker, W. (1982). Physical fitness training and counseling as treatment for youthful offenders. *Journal of Counseling Psychology*, *29*(3), 292.

Hindman, R., Glass, C., Arnkoff, D., & Maron, D. (2014). A Comparison of Formal and Informal Mindfulness Programs for Stress Reduction in University Students. *Mindfulness*, *6*. <https://doi.org/10.1007/s12671-014-0331-1>

Kenardy, J., McCafferty, K., & Rosa, V. (2003). Internet-Delivered Indicated Prevention For Anxiety Disorders: A Randomized Controlled Trial. *Behavioural and Cognitive Psychotherapy*, *31*. <https://doi.org/10.1017/S1352465803003047>

Khalsa, S. B., Hickey-Schultz, L., Cohen, D., Steiner, N., & Cope, S. (2012). Evaluation of the mental health benefits of yoga in a secondary school: a preliminary randomized controlled trial. *J Behav Health Serv Res*, *39*(1), 80-90. <https://doi.org/10.1007/s11414-011-9249-8>

Levin, M. E., Haeger, J. A., Pierce, B. G., & Twohig, M. P. (2017). Web-Based Acceptance and Commitment Therapy for Mental Health Problems in College Students: A Randomized Controlled Trial. *Behav Modif*, *41*(1), 141-162. <https://doi.org/10.1177/0145445516659645>

Levin, M. E., Hayes, S. C., Pistorello, J., & Seeley, J. R. (2016). Web-Based Self-Help for Preventing Mental Health Problems in Universities: Comparing Acceptance and Commitment Training to Mental Health Education. *J Clin Psychol*, *72*(3), 207-225. <https://doi.org/10.1002/jclp.22254>

Levin, M. E., Navarro, C., Cruz, R. A., & Haeger, J. (2019). Comparing in-the-moment skill coaching effects from tailored versus non-tailored acceptance and commitment therapy mobile apps in a non-clinical sample. *Cogn Behav Ther*, *48*(3), 200-216. <https://doi.org/10.1080/16506073.2018.1503706>

MacMahon, J. R., & Gross, R. T. (1988). Physical and psychological effects of aerobic exercise in delinquent adolescent males. *Am J Dis Child*, *142*(12), 1361-1366. <https://doi.org/10.1001/archpedi.1988.02150120115053>

McGrady, A., Brennan, J., Lynch, D., & Whearty, K. (2012). A wellness program for first year medical students. *Appl Psychophysiol Biofeedback*, *37*(4), 253-260. <https://doi.org/10.1007/s10484-012-9198-x>

Melnyk, B. M., Jacobson, D., Kelly, S., O'Haver, J., Small, L., & Mays, M. Z. (2009). Improving the mental health, healthy lifestyle choices, and physical health of Hispanic adolescents: a randomized controlled pilot study. *J Sch Health*, *79*(12), 575-584. <https://doi.org/10.1111/j.1746-1561.2009.00451.x>

Merry, S. N., Stasiak, K., Shepherd, M., Frampton, C., Fleming, T., & Lucassen, M. F. G. (2012). The effectiveness of SPARX, a computerised self help intervention for adolescents seeking help for depression: randomised controlled non-inferiority trial. *BMJ : British Medical Journal*, *344*, e2598. <https://doi.org/10.1136/bmj.e2598>

Moir, F., Henning, M., Hassed, C., Moyes, S. A., & Elley, C. R. (2016). A Peer-Support and Mindfulness Program to Improve the Mental Health of Medical Students. *Teach Learn Med*, *28*(3), 293-302. <https://doi.org/10.1080/10401334.2016.1153475>

Nabkasorn, C., Miyai, N., Sootmongkol, A., Junprasert, S., Yamamoto, H., Arita, M., & Miyashita, K. (2006). Effects of physical exercise on depression, neuroendocrine stress hormones and physiological fitness in adolescent females with depressive symptoms. *Eur J Public Health*, *16*(2), 179-184. <https://doi.org/10.1093/eurpub/cki159>

Nguyen-Feng, V., Frazier, P., Greer, C., Meredith, L., Howard, K., & Paulsen, J. (2016). Testing the efficacy of three brief web-based interventions for reducing distress among interpersonal violence survivors. *Translational Issues in Psychological Science*, *2*, 439-448. <https://doi.org/10.1037/tps0000099>

Nguyen-Feng, V. N., Greer, C. S., & Frazier, P. (2017). Using online interventions to deliver college student mental health resources: Evidence from randomized clinical trials. *Psychol Serv*, *14*(4), 481-489. <https://doi.org/10.1037/ser0000154>

Nguyen-Feng, V. N., Romano, F. N., & Frazier, P. (2019). Emotional abuse moderates efficacy of an ecological momentary stress management intervention for college students. *J Couns Psychol*, *66*(4), 461-472. <https://doi.org/10.1037/cou0000332>

Norris, R., Carroll, D., & Cochrane, R. (1992). The effects of physical activity and exercise training on psychological stress and well-being in an adolescent population. *J Psychosom Res*, *36*(1), 55-65. <https://doi.org/10.1016/0022-3999(92)90114-h>

Phang, C. K., Mukhtar, F., Ibrahim, N., Keng, S. L., & Mohd Sidik, S. (2015). Effects of a brief mindfulness-based intervention program for stress management among medical students: the Mindful-Gym randomized controlled study. *Adv Health Sci Educ Theory Pract*, *20*(5), 1115-1134. <https://doi.org/10.1007/s10459-015-9591-3>

Putra, E. S., Wasita, B., & Anantanyu, S. (2018). A randomised trial on walking exercise and banana consumption on self-reported depression symptoms among female adolescents in Surakarta, Indonesia. *Malaysian Journal of Nutrition*, *24*(3), 467-473.

Raes, F., Griffith, J., van der gucht, K., & Williams, J. (2014). School-Based Prevention and Reduction of Depression in Adolescents: a Cluster-Randomized Controlled Trial of a Mindfulness Group Program. *Mindfulness*, *5*. <https://doi.org/10.1007/s12671-013-0202-1>

Rentala, S., Thimmajja, S. G., Tilekar, S. D., Nayak, R. B., & Aladakatti, R. (2019). Impact of holistic stress management program on academic stress and well-being of Indian adolescent girls: A randomized controlled trial. *J Educ Health Promot*, *8*, 253. <https://doi.org/10.4103/jehp.jehp_233_19>

Reynolds, W. M., & Coats, K. I. (1986). A comparison of cognitive-behavioral therapy and relaxation training for the treatment of depression in adolescents. *J Consult Clin Psychol*, *54*(5), 653-660. <https://doi.org/10.1037//0022-006x.54.5.653>

Robledo-Colonia, A. F., Sandoval-Restrepo, N., Mosquera-Valderrama, Y. F., Escobar-Hurtado, C., & Ramírez-Vélez, R. (2012). Aerobic exercise training during pregnancy reduces depressive symptoms in nulliparous women: a randomised trial. *J Physiother*, *58*(1), 9-15. <https://doi.org/10.1016/s1836-9553(12)70067-x>

Roth, D. L. (1989). Acute emotional and psychophysiological effects of aerobic exercise. *Psychophysiology*, *26*(5), 593-602. <https://doi.org/10.1111/j.1469-8986.1989.tb00716.x>

Roth, D. L., & Holmes, D. S. (1987). Influence of aerobic exercise training and relaxation training on physical and psychologic health following stressful life events. *Psychosom Med*, *49*(4), 355-365. <https://doi.org/10.1097/00006842-198707000-00004>

Sagon, A., Danitz, S., Suvak, M., & Orsillo, S. (2018). The Mindful Way through the Semester : Evaluating the Feasibility of Delivering an Acceptance-Based Behavioral Program Online. *Journal of Contextual Behavioral Science*, *9*. <https://doi.org/10.1016/j.jcbs.2018.06.004>

Saravanan, C., & Kingston, R. (2014). A randomized control study of psychological intervention to reduce anxiety, amotivation and psychological distress among medical students. *J Res Med Sci*, *19*(5), 391-397.

Scholten, H., Malmberg, M., Lobel, A., Engels, R. C., & Granic, I. (2016). A Randomized Controlled Trial to Test the Effectiveness of an Immersive 3D Video Game for Anxiety Prevention among Adolescents. *PLoS One*, *11*(1), e0147763. <https://doi.org/10.1371/journal.pone.0147763>

Seligman, M., Schulman, P., DeRubeis, R., Hollon, S., Balonek, A., Berland, B., Blaney, B., Brannon, E., Bromley, S., Khanna, M., Clark, D., Colton, K., Dauman, R., Drummer, L., Eisenberg, A., Epstein, T., Estep, A., Fink, M., Fishback, J., & Rubenstein, Y. (2000). The prevention of depression and anxiety. *2*.

Seligman, M. E., Schulman, P., & Tryon, A. M. (2007). Group prevention of depression and anxiety symptoms. *Behav Res Ther*, *45*(6), 1111-1126. <https://doi.org/10.1016/j.brat.2006.09.010>

Shapiro, S. L., Schwartz, G. E., & Bonner, G. (1998). Effects of mindfulness-based stress reduction on medical and premedical students. *J Behav Med*, *21*(6), 581-599. <https://doi.org/10.1023/a:1018700829825>

Shearer, A., Hunt, M., Chowdhury, M., & Nicol, L. (2015). Effects of a Brief Mindfulness Meditation on Student Stress and Heart Rate Variability. *International Journal of Stress Management*, *23*. <https://doi.org/10.1037/a0039814>

Song, Y., & Lindquist, R. (2015). Effects of mindfulness-based stress reduction on depression, anxiety, stress and mindfulness in Korean nursing students. *Nurse Educ Today*, *35*(1), 86-90. <https://doi.org/10.1016/j.nedt.2014.06.010>

Stasiak, K., Hatcher, S., Frampton, C., & Merry, S. N. (2014). A pilot double blind randomized placebo controlled trial of a prototype computer-based cognitive behavioural therapy program for adolescents with symptoms of depression. *Behav Cogn Psychother*, *42*(4), 385-401. <https://doi.org/10.1017/s1352465812001087>

Ștefan, C. A., Căpraru, C., & Szilágyi, M. (2018). Investigating effects and mechanisms of a mindfulness-based stress reduction intervention in a sample of college students at risk for social anxiety. *Mindfulness*, *9*(5), 1509-1521.

Van Aubel, E., Bakker, J. M., Batink, T., Michielse, S., Goossens, L., Lange, I., Schruers, K., Lieverse, R., Marcelis, M., & van Amelsvoort, T. (2020). Blended care in the treatment of subthreshold symptoms of depression and psychosis in emerging adults: a randomised controlled trial of acceptance and commitment therapy in daily-life (ACT-DL). *Behaviour research and therapy*, *128*, 103592.

Vázquez, F. L., Torres, A., Blanco, V., Díaz, O., Otero, P., & Hermida, E. (2012). Comparison of relaxation training with a cognitive-behavioural intervention for indicated prevention of depression in university students: a randomized controlled trial. *J Psychiatr Res*, *46*(11), 1456-1463. <https://doi.org/10.1016/j.jpsychires.2012.08.007>

Velasquez, A., López, M., Quiñonez, N., & Paba, D. (2015). Yoga for the prevention of depression, anxiety, and aggression and the promotion of socio-emotional competencies in school-aged children. *Educational Research and Evaluation*, *21*, 1-15. <https://doi.org/10.1080/13803611.2015.1111804>

Walsh, E., Eisenlohr-Moul, T., & Baer, R. (2016). Brief mindfulness training reduces salivary IL-6 and TNF-α in young women with depressive symptomatology. *J Consult Clin Psychol*, *84*(10), 887-897. <https://doi.org/10.1037/ccp0000122>

Warnecke, E., Quinn, S., Ogden, K., Towle, N., & Nelson, M. R. (2011). A randomised controlled trial of the effects of mindfulness practice on medical student stress levels. *Med Educ*, *45*(4), 381-388. <https://doi.org/10.1111/j.1365-2923.2010.03877.x>

Zhang, J., Qin, S., Zhou, Y., Meng, L., Su, H., & Zhao, S. (2018). A randomized controlled trial of mindfulness-based Tai Chi Chuan for subthreshold depression adolescents. *Neuropsychiatr Dis Treat*, *14*, 2313-2321. <https://doi.org/10.2147/ndt.S173255>
